# It’s not all in your feet: Improving penalty kick performance with human-avatar interaction and machine learning

**DOI:** 10.1016/j.xinn.2024.100584

**Published:** 2024-02-06

**Authors:** Jean-Luc Bloechle, Julien Audiffren, Thibaut Le Naour, Andrea Alli, Dylan Simoni, Gabriel Wüthrich, Jean-Pierre Bresciani

**Affiliations:** 1 Control and Perception Laboratory, University of Fribourg, Bd Perolles 90, 1700 Fribourg, Switzerland; 2 Motion-up, Le Prisme, Place Albert Einstein, 56000 Vannes, France; 3 FC Basel 1893, Birsstrasse 320A, 4002 Basel, Switzerland

## Abstract

Penalty kicks are increasingly decisive in major international football competitions. Yet, over 30% of shootout kicks are missed. The outcome of the kick often relies on the ability of the penalty taker to exploit anticipatory movements of the goalkeeper to redirect the kick toward the open side of the goal. Unfortunately, this ability is difficult to train using classical methods. We used an augmented reality simulator displaying an holographic goalkeeper to test and train penalty kick performance with 13 young elite players. Machine learning algorithms were used to optimize the learning rate by maintaining an optimal level of training difficulty. Ten training sessions of 20 kicks reduced the redirection threshold by 120 ms, which constituted a 28% reduction with respect to the baseline threshold. Importantly, redirection threshold reduction was observed for all trained players, and all things being equal, it corresponded to an estimated 35% improvement of the success rate.

## Public summary

• In major football competitions, over 20% of knockout games are decided by penalty shootouts.
• Thirty percent of the kicks are missed, notably because players lack specific and adapted training methods.
• We developed an augmented reality simulator with a holographic goalkeeper to train sensorimotor kicking skills.
• Ten sessions of machine learning-optimized training improved the sensorimotor skills of players by 28%.
• This translates into a 35% increase of the success rate, thereby constituting a powerful training tool.

## Graphical abstract

**Figure:**
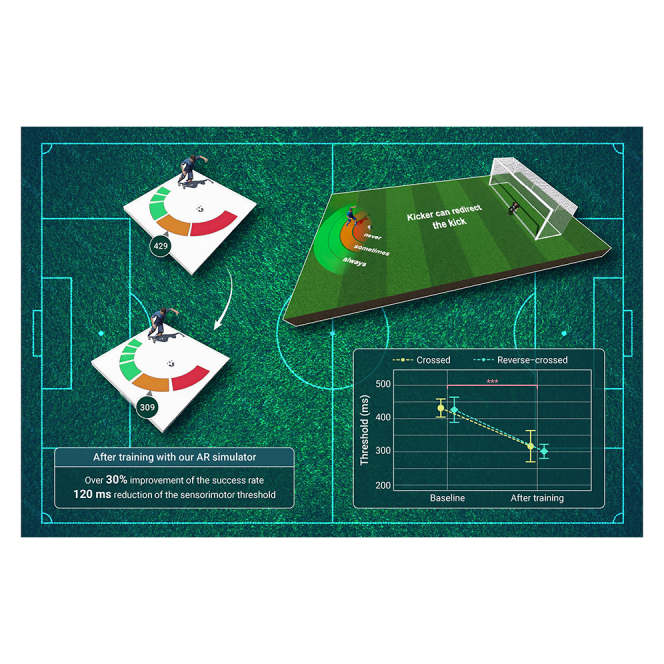

## Introduction

In the last 40 years, penalty kicks have often been decisive in international football competitions.^1^ In the knockout phases of the FIFA World Cup, 21% of the games were decided by penalty shootouts, as was the final of the recent 2022 World Cup. This number reaches 28% regarding UEFA Champions League finals. In shootout sessions, about 30% of the penalties are missed.^2^^,^^3^ Such a high miss rate is surprising when considering the advantage of the player over the goalkeeper. Indeed, the goalkeeper can only cover a small portion of the 18 square meters goal area. In addition, the goalkeeper needs about 900 ms to dive and reach a side of the goal (ie, 200 ms of visual reaction time^4^ and 700 ms of movement/diving time^5^), whereas the ball reaches the goal less than 500 ms after the kick.^6^^,^^7^ If the player shoots to a side of the goal, the goalkeeper must start moving at least 300 ms before foot-ball contact to stand a chance to block the kick. And indeed, professional goalkeepers anticipate-dive to a side of the goal in about 95% of penalty kicks.^8^ Consequently, penalty takers developed a strategy consisting in “awaiting” an early dive of the goalkeeper that would allow them to kick the ball to the “open” side of the goal.^3^ Specifically, the player selects a side in advance, but this plan is subject to alterations depending on the goalkeeper’s movements.^9^^,^^10^^,^^11^^,^^12^^,^^13^^,^^14^^,^^15^ This strategy, called goalkeeper dependent, is adopted by 75% of professional penalty takers.^3^ With this strategy, three scenarios are possible. If the goalkeeper does not move before foot-ball contact, the player kicks toward the initially selected side. If, before foot-ball contact, the goalkeeper dives to the side opposite the one selected by the player, the player also kicks toward the initially selected side. If, however, the goalkeeper dives (before foot-ball contact) to the side initially selected by the player, this latter must modify his/her motor plan during the run-up to kick the ball toward the open side of the goal. Unfortunately, redirecting the kick is not always possible. In particular, the sensorimotor loops underlying kick redirection require time to process visual information relative to the goalkeeper and to modify the initial motor plan. Put differently, successfully redirecting the kick to score the penalty is only possible if there is enough time left before foot-ball contact.

The minimum time needed to successfully modify an ongoing movement, the “new” movement accurately corresponding to the desired outcome, has been extensively studied. Most of these studies are based on arm reaching movements, and perturbations are introduced during the movement.^16^^,^^17^^,^^18^^,^^19^^,^^20^^,^^21^^,^^22^^,^^23^^,^^24^ Visually driven corrections of such reaching movements are efficient, smooth, and occur with short latency. Deviations of the hand trajectory are usually observed between 280 and 350 ms after perturbation,^17^^,^^25^^,^^26^ although under certain conditions, they can occur as early as 130 ms after perturbation.^18^ Interestingly, when measured in comparable conditions and with similar tasks, online modifications occur almost twice faster as visual reaction times.^19^ Therefore, many authors have suggested that as opposed to typical reaction times, online modifications are largely automatic^16^^,^^17^^,^^18^^,^^27^ and could rely on subcortical control loops.^19^ Unfortunately, in contrast with the abundant literature describing the characteristics and efficiency of the sensorimotor loops controlling simple reaching movements, little is known about the online control of more complex movements. This is notably because complex movements are harder to study in a controlled fashion.

**Figure 1:**
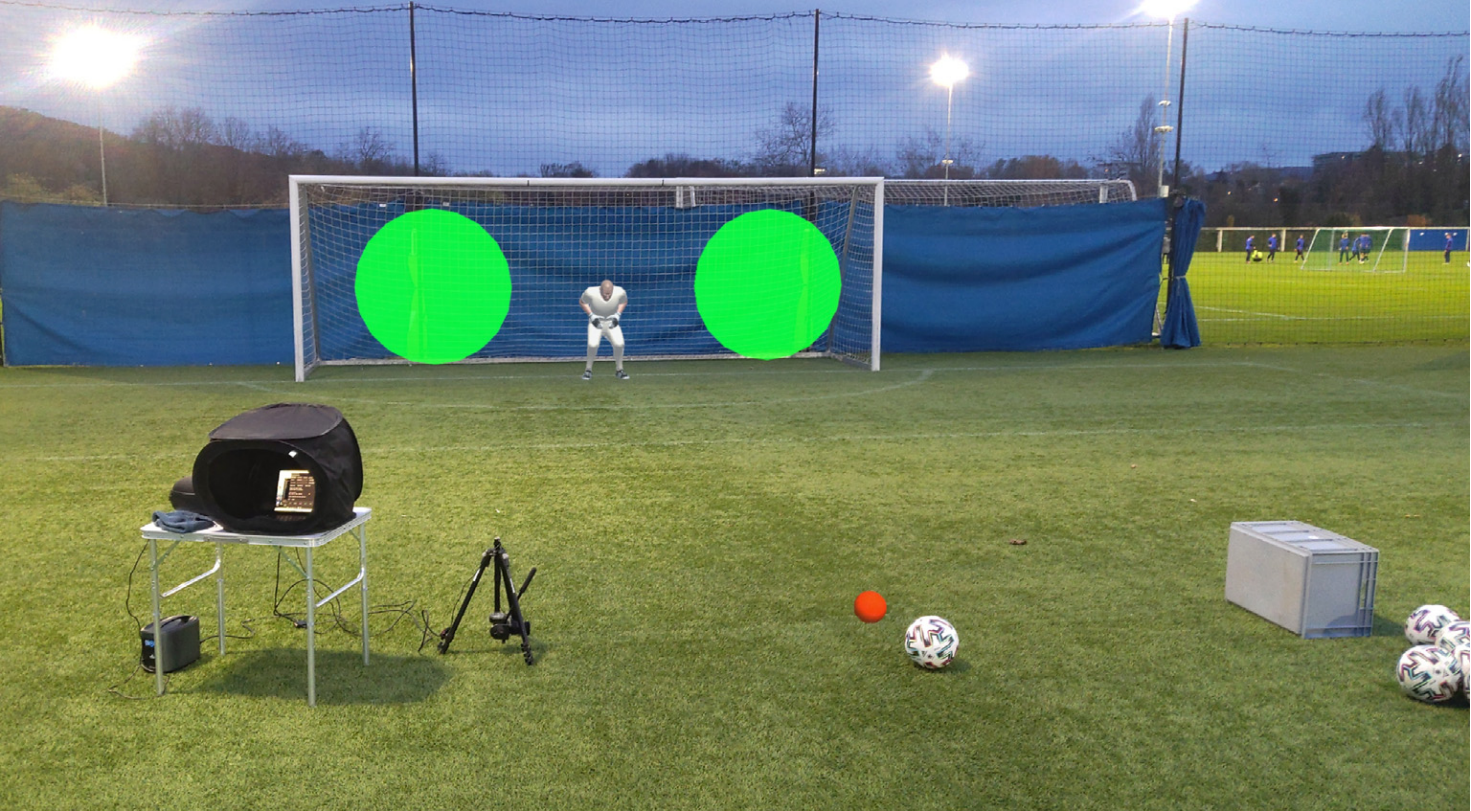
Visual scene viewed through a Microsoft HoLolens 2 headset The player’s view after the ball has been positioned on the penalty mark. The red holographic sphere indicates the player’s gaze location.

**Figure 2:**
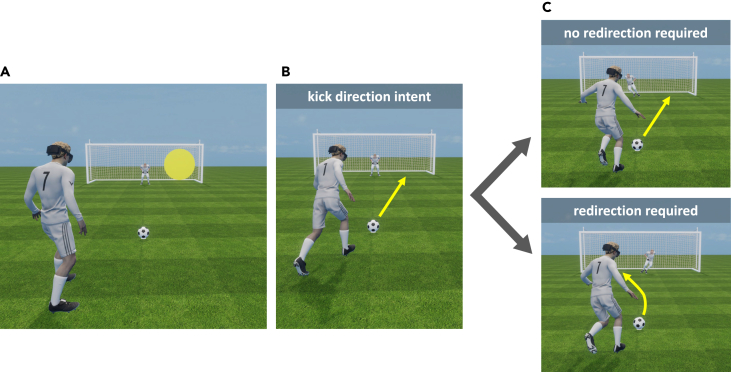
The different stages of a trial for the penalty taker (A) The player is about to start running up. The yellow target indicates where to kick the ball. (B) The player starts running up to the ball with the “objective” to kick the ball to the previously displayed target area. (C) Upper panel: no redirection trial—during the run-up, the holographic goalkeeper dives to the side opposite the “target side”; no kick redirection is required. Lower panel: redirection trial—the holographic goalkeeper dives to the target side; the penalty taker must change his motor plan and redirect the kick toward the open side of the goal.

Here, we quantified the minimum time necessary to successfully redirect a penalty kick, and more importantly, we assessed whether this time can be “shortened” using appropriate training. We modified the double-step paradigm traditionally used to study the online control of reaching movements, and adapted it to a realistic penalty kick simulation. Specifically, we developed an augmented reality simulator in which football players tried to score penalty kicks on a real soccer pitch, with a real soccer ball and a real goal, but facing a human-like holographic goalkeeper (see Figure 1). During the run-up to the ball, the holographic goalkeeper dove to one side of the goal as real goalkeepers anticipate-dive. For half of the kicks, the dive forced the penalty taker to redirect the kick (see Figure 2). For each player and each trial, the dive onset depended on the estimated time to foot-ball contact. This time was estimated using a time/radius mapping algorithm based on kinematic information relative to both the current and previous run-ups to the ball. Dive onset was adjusted from trial to trial as a function of two factors, namely (1) the actual time of the dive before foot-ball contact (as measured on previous trials) and (2) the associated performance of the penalty taker, ie, his sensorimotor ability to successfully redirect the kick when needed. As this sensorimotor ability improved, the task became more and more difficult. Put differently, as the minimum time required by the player to successfully redirect the kick decreased, the goalkeeper dive occurred later in the run-up to the ball, which made it harder for the player to redirect the kick. A Bayesian network was used to model the player’s current level of performance and its evolution in order to adjust the difficulty of the task and optimize the training rate. This optimization aimed at bringing each player to the best possible performance in the minimum training time.

## Results

The first two sessions were used to estimate the baseline performance of each player, namely the 50% redirection threshold. On average, the baseline redirection threshold was 429.02 ms (±53.46, range: 319–536 ms). After 10 training sessions, the 50% redirection threshold dropped to 309.08 ms (±59.76, range: 213–488 ms). This 120 ms threshold reduction was significant ($\chi^{2}\left(1\right)=44.15$, p<0.001), it explained 54% of the variance (marginal $R^{2}=0.54$), and it constituted a 28% reduction as compared with baseline.

**Figure 3:**
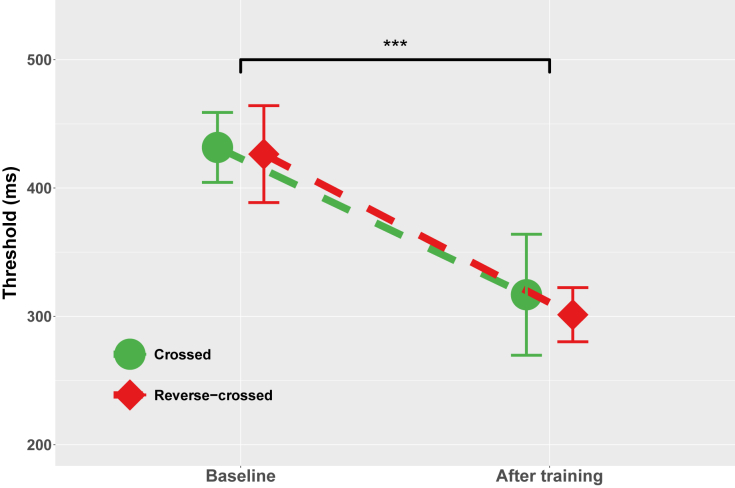
Average redirection threshold before (baseline) and after training The redirection threshold was significantly lower after training (p < 0.001). The pattern is similar for crossed (green, redirection toward the side opposite the kicking foot) and reverse-crossed redirection (red, redirection toward the side of the kicking foot). The error bars represent the 95% confidence interval.

**Figure 4:**
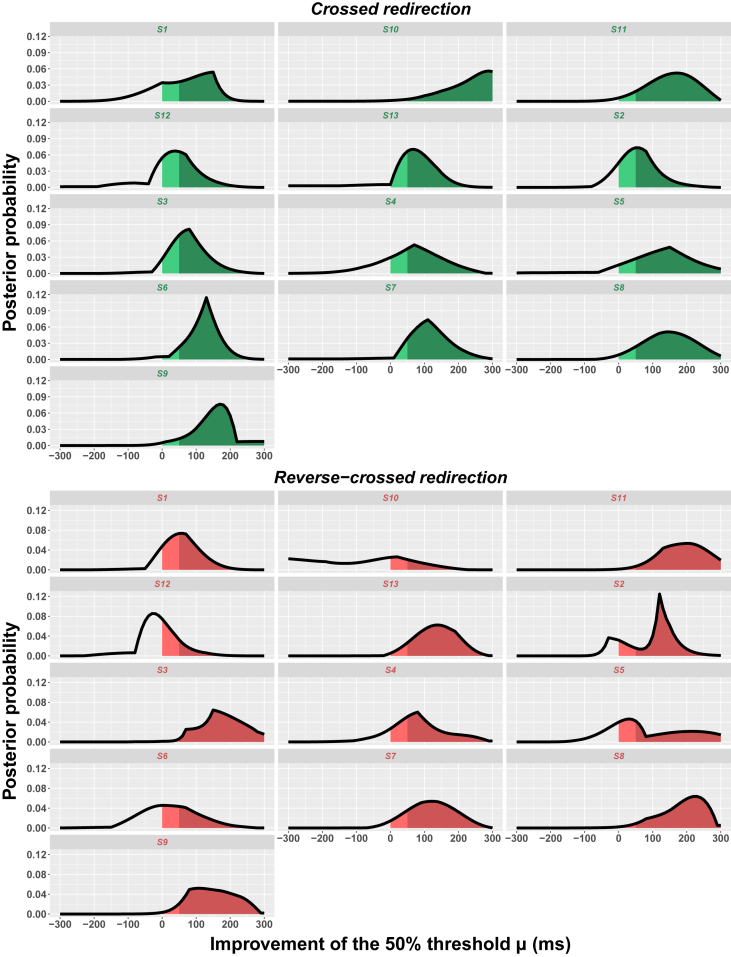
Probability of improvement of the redirection threshold for crossed (green) and reverse-crossed (red) redirection For each player (S*i*), the curve represents the estimated distribution of the redirection threshold after training (relative to baseline performance). The area under the curve for X values larger than 0 (i.e., green-shaded area for crossed redirection and red-shaded area for reverse-crossed redirection) shows the probability of improvement of the redirection threshold, i.e., the probability that the redirection threshold be lower after training. The dark-shaded area corresponds to the probability that the improvement be larger than one standard deviation.

We then assessed whether and how the side of the required redirection affected the redirection threshold. When the player had to redirect the kick toward the side opposite the kicking foot (ie, redirection to the left for a right-footed player), redirection was defined as crossed redirection. When the player had to redirect the kick toward the side of the kicking foot (ie, redirection to the right for a right-footed player), redirection was defined as reverse-crossed redirection.^28^ The redirection threshold was not affected by the redirection side ($\chi^{2}\left(1\right)=0.61$, p=0.43), and there was no interaction between the session and the redirection side ($\chi^{2}\left(1\right)=0.15$, p=0.70). The redirection side did not affect either the baseline threshold (431.62 ± 45.05 ms vs. 426.41 ± 62.52 ms, p=0.68, Bayes factor = 0.29) or the training-evoked threshold reduction (−114.77 ± 78.72 ms vs. −125.10 ± 46.93 ms, p=0.79, Bayes factor = 0.31). Importantly, as shown in Figure 3, a significant threshold reduction was observed after training for both crossed (p<0.01, R=0.82) and reverse-crossed redirection (p<0.001, R=0.88). Figure 4 shows the probability of reduction of the redirection threshold as a function of the reduction amplitude (in ms) for each of the players and the two redirection sides. This probability (derived by our Bayesian network) ranged from 0.29 to 0.99 (mean = 0.84 ± 0.17, median = 0.88), and all values but two were larger than 0.7, indicating a high probability of improvement. The probability of observing such an outcome by chance, namely 24 improvements out of 26 draws, is about 1/100,000 (as assessed by a two-tailed binomial test). We also computed the probability that the training-evoked improvement be larger than one standard deviation. This probability ranged from 0.16 to 0.94 (mean = 0.7 ± 0.23, median = 0.73), with all values but four above 0.5.

**Figure 5:**
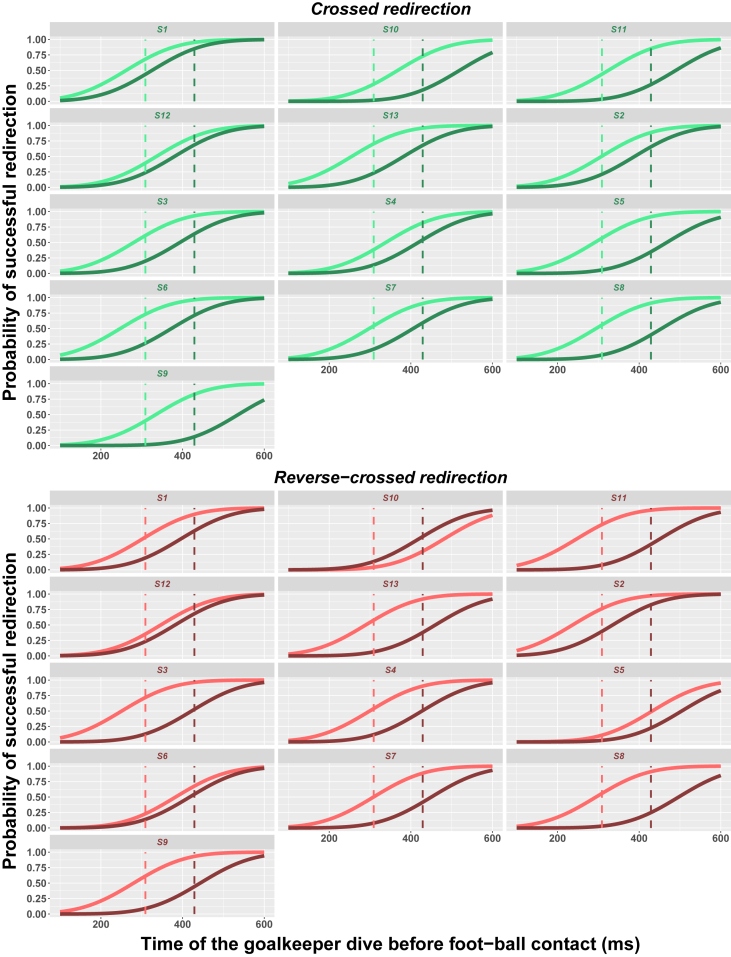
Probability of successful redirection for crossed (green) and reverse-crossed (red) redirection The curves represent the estimated probability of successfully redirecting the kick as a function of the time of the goalkeeper dive before foot-ball contact. For each player (S*i*), the dark-colored curve shows the estimated baseline probability, whereas the light-colored curve shows the estimated probability after training. For any X value, the Y value difference between the two curves corresponds to the training-evoked change of probability. The light-colored curve is almost always above the dark-colored curve, indicating an increase of the probability to successfully redirect the kick after training. The dashed vertical lines indicate the average (population-wise) redirection threshold before (dark colored) and after training (light colored).

From an applied perspective, what coaches and football professionals probably want to know is how the redirection threshold reduction translates in terms of success rate. When taking the baseline redirection threshold as reference performance (ie, 429 ms), the training-evoked threshold reduction corresponds to an estimated 34% improvement of the success rate. The estimated success rate rises from 49% ± 21% and 52% ± 18% before training (for crossed and reverse-crossed redirection, respectively) to 89% ± 7% and 81% ± 21% after training. The before vs. after difference is significant in both cases (p<0.001, R=0.88 and p<0.01, R=0.80). When taking the redirection threshold measured after training as reference (ie, 309 ms), the average improvement is 36%, and the estimated success rate rises from 14% ± 12% and 15% ± 10% before training to 53% ± 14% and 47% ± 23% after training. Again, the before vs. after difference is significant in both cases (p<0.001, R=0.88 and p<0.01, R=0.83). Figure 5 shows the effect of training on the estimated probability of successful redirection as a function of time.

## Discussion

Only 10 sessions of 20 kicks with our simulator resulted in a 120 ms (ie, 28%) reduction of the redirection threshold. All things being equal, this threshold reduction translates into a sizable 35% improvement of the success rate. Importantly, the probability of the training to reduce the redirection threshold was 84% on average, and superior to 70% in 24 of the 26 player-side combinations. Similarly, the training substantially increased the probability of success rate for 23 of the 26 player-side combinations, and this over a large time range of anticipation-dive of the goalkeeper.

Very few studies previously attempted to address the control and redirection of penalty kicks.^29^^,^^30^ In these studies, the approach to the problem was different from ours, and penalty simulations were non-realistic. Specifically, one study^29^ measured choice reaction times (lever-tilting task), which are different from online responses.^19^^,^^23^^,^^31^ The other study^30^ used light bulbs instead of a goalkeeper, which can alter the player’s behavior^32^ and attention orienting processes.^33^ In addition, both studies exclusively investigated redirection thresholds. As opposed to that, we combined augmented reality, human-avatar interaction and machine learning algorithms to develop an ecologically valid simulation allowing us to reduce the redirection threshold and improve success rate. Our simulator is used on a soccer pitch with soccer balls, and the visual stimulus triggering the redirection response is an holographic goalkeeper having the same size and moving exactly as a real goalkeeper, thereby matching realistically the real penalty kick situation. Those factors confer physical, biomechanical, and perceptive-cognitive fidelity to our simulator,^34^^,^^35^^,^^36^ making the task at hand more engaging^37^^,^^38^ and increasing transfer likelihood.^39^^,^^40^^,^^41^^,^^42^ Finally, our Bayesian network grants a more accurate and reliable estimation of the minimum time required to successfully redirect the kick. Specifically, both anticipation behavior and the “global” rate of failed kicks (ie, the proportion of failed kicks that are not imputable to the redirection constraint) are taken into account to limit anticipation-related bias when estimating the threshold value.^43^^,^^44^^,^^45^ Therefore, our Bayesian network allows us to finely model the individual performance of each player (see Figures S4 and S5), and the player model is continuously updated by integrating the performance on the “new” trials (see Figure S6).

As mentioned above, an important proportion of games in international football competitions are decided by penalty shootouts. These sessions have a “dramatic flavor,” both for the teams and their supporters. Therefore, the fear of missing puts a lot of pressure and stress on the penalty taker, especially when he/she has a lot riding on his/her kick.^46^^,^^47^^,^^48^^,^^49^^,^^50^ This psychological pressure has a negative impact on the success rate.^51^ Being well prepared and more aware of your skills is probably one of the most efficient ways to cope with such stressful situations.^52^^,^^53^ Accordingly, the ability to train players to successfully redirect the kick later in the run-up will not only increase their success rate, but will also contribute to reduce their stress.^54^ In that respect, our simulator constitutes a unique training tool allowing players to practice penalty kicks and improve their sensorimotor skills in a way that would be impossible otherwise. In particular, the simulator precisely triggers the dive of the goalkeeper based on the run-up of the player, which would be impossible with a real goalkeeper. Coupled with our optimization algorithms, this grants the possibility to permanently adjust the difficulty of the training to keep the athlete in the “optimal challenge zone” (ie, neither too easy nor too difficult). This maintains the athlete at a high level of motivation and optimizes his/her learning pace.^55^^,^^56^ In addition, the virtual goalkeeper can perform an infinite number of successive dives without risking any injury, which would be impossible with a real goalkeeper. Thus, our simulator provides an unparalleled tool to flexibly organize targeted training sessions.

As a final thought, we should mention that although the sensorimotor skills trained with our simulator seem very specific, they are not. Specifically, being able to redirect a penalty kick based on the visually detected movements of the goalkeeper is very similar to being able to redirect a pass based on the perceived movements of teammates and opponents. In that respect, we believe that the sensorimotor skills trained with our simulator would transfer, at least to some extent, to all game situations in which the player should pass/kick the ball under time constraint, eg, when pressed by a direct opponent or when about to pass the ball to a partner who is now marked or has changed position. Almost every time a player passes the ball, there is more than one passing option. The “best” option quickly changes because partners and opponents are constantly moving. Being able to redirect the kick shortly before kicking the ball increases the chances to deliver the ball to the best positioned partner at this very moment. This applies to all players on the pitch. Slight modifications to our simulator will grant the possibility to manipulate and control all relevant factors with precise timing to optimize progress rate. Therefore, the approach developed in this study should find larger applications, be it in football or other sports in which being able to modify the planned/ongoing action as late as possible provides a decisive advantage as, for instance, tennis or ice hockey.

## Materials and methods

### Participants

Thirteen young elite football players (U16 to U18 from FC Luzern and FC Basel) participated in the experiment (mean age = 15.77 ± 0.73; age range: 15–17; 13 male, 5 left-footed). Eight of them played for the U15, U16, and/or U17 Swiss national team. All were able-bodied with normal or corrected-to-normal vision. The study was performed in accordance with the ethical standards laid down in the 1964 Declaration of Helsinki and approved by the Ethics Committee of the University of Fribourg. Participants had the option to withdraw from the study at any time without penalty and without having to give a reason.

### Setup/apparatus

The experiment was performed in the penalty area of a football pitch (grass), with a football goal and official match balls. The players were dressed in football outfits, and wore a Microsoft HoloLens 2 headset,^57^ which is a “see-through” augmented reality headset. The headset was used to display the virtual part of the visual scene. When the luminosity was very high, a homemade filter (dark plastic film) was applied on the headset to increase the contrast of the virtual scene. The headset was also used to track the position of the player on the pitch. A LIDAR sensor (TeraRanger Evo 60m, sampling frequency of 240Hz, USB connection) fixed on a tripod was positioned to the side of the ball at a 1.5-m distance. The LIDAR was always positioned on the kicking foot side, and it was used to time and record foot-ball contact. A gray box opposite the LIDAR (2 m from the ball) reflected the laser beam after the kick. A laptop used to run the experiment and a mobile phone used as Wi-Fi hotspot (network communication between the laptop and the headset) were on a table next to the LIDAR. A portable PowerStation ensured the electric charging of all devices.

### Virtual scene and holographic goalkeeper

The virtual scene was displayed in the headset. It consisted of the holographic goalkeeper and of colored 3D spheres and 2D target areas, depending on the phase of the trial (see procedure). The SimulKick application (see supplemental information) managed the scene and animated the holographic goalkeeper in real time, notably triggering his dives. The animations were based on the motion-captured movements of a professional goalkeeper (see supplemental information).

### Procedure

At the beginning of each trial, the holographic goalkeeper was in the middle of the goal. The player had to put the ball on the penalty mark, which was highlighted by a red holographic sphere. This sphere turned green once the ball was on the mark. Two holographic target areas (2D disc, diameter 200 cm/10.4° of visual angle in diameter) were then displayed in red next to the left and right posts. Once the player was in his “starting” position for the run-up (at least 2 m from the ball), the two holographic targets turned green (see Figure 1). Before starting running up, the player had to fixate the goalkeeper’s head for 3 s. One of the targets was then switched off and the other one turned yellow. The yellow target indicated where to kick the ball (left or right side of the goal, see Figure S1) and the player could initiate the run-up. The yellow target was switched off during the run-up, when the player was at a 2-m distance from the ball. During the run-up, the holographic goalkeeper dove to one side of the goal (left or right, see Figure S2). When the goalkeeper dove to the side opposite the previously displayed yellow target, no kick redirection was required. When the goalkeeper dove to the side where the yellow target was previously lit, the player had to redirect the kick toward the other side of the goal (opposite the initial target position). In other words, the player always had to kick the ball toward the “open side” of the goal. Figure 2 summarizes the kicking options for the player. The kick was successful when the player redirected the kick without anticipating redirection (see supplemental information). Figure S3 shows a player about to kick the ball. After the dive and the kick, the goalkeeper walked back to the center of the goal. The goalkeeper was displayed for the whole duration of the session, with different animations depending on the “stage” of the penalty kick (ie, before, during, or after the run-up). After each kick, the actual time of the goalkeeper dive and the success of the kick were registered in KickManager (see supplemental information). Each training session consisted of 20 penalty kicks, for a total duration of 15 min per session. Within any given session, the initial target side was always the same (right or left). Out of the 20 kicks, 12 randomly selected kicks required redirection (60% of the kicks). Each player performed two to three training sessions per week, and there was never more than one training session per day.

### Triggering of the goalkeeper dive

The goalkeeper dive was triggered by SimulKick during the player’s run-up. The onset of the dive could change from trial to trial based on (1) the estimated time before foot-ball contact and (2) the estimated level of performance of the player at this stage of the training (see supplemental information).

### Data analysis

The session (baseline vs. after training) and the direction (crossed vs. reverse-crossed) of the required redirection were within subject factors (repeated measures). The dependent variable was the 50% redirection threshold, namely the minimum time required to successfully redirect the kick 50% of the time. The effect of the two factors and their interaction on the dependent variable was assessed using a linear mixed-effects modeling approach (see supplemental information). For each factor, the effect size was computed using the marginal $R^{2}$. Direct comparisons between means were performed using Wilcoxon signed-rank tests for repeated measures, and the effect size was computed using Pearson’s R. When the Wilcoxon test was non-significant, we additionally computed the Bayes factor.

## Supplemental information

Document S1. Figures S1–S6

Document S2. Article plus supplemental information
