# Supplementary material for: It’s not all in your feet: Improving penalty kick performance with human-avatar interaction and machine learning
Source: Innovation (Camb). 2024 Feb 6;5(2):100584. doi: 10.1016/j.xinn.2024.100584 (PMC10912701; doi:10.1016/j.xinn.2024.100584)
Supplement: Document S1. Figures S1–S6 [file mmc1.pdf]

The Innovation, Volume *5*

Supplemental Information

It's not all in your feet: Improving penalty kick performance with human-avatar interaction and machine learning

Jean-Luc Bloechle, Julien Audiffren, Thibaut Le Naour, Andrea Alli, Dylan Simoni, Gabriel Wüthrich, and Jean-Pierre Bresciani

Supplemental material

The SimulKick application

SimulKick was developed in the C# programming language with the Unity 3D real-time engine and the Microsoft's MRTK augmented reality toolkit. The frequency of the physics engine of SimulKick was set to 100 Hz. During the experiment, SimulKick continuously communicated with the KickManager application via Wi-Fi (using UDP communication together with the JSON data-interchange format). KickManager was developed in the Java programming language, and used by the operator to manage player sessions and upload them to SimulKick. The KickManager application was also used to transmit real-time data from the LIDAR to the SimulKick application, in particular to indicate whether the ball was on the penalty mark. Both the SimulKick and the KickManager applications were developed in our lab.

Animation of the holographic goalkeeper

To create the animations of the holographic goalkeeper, a goalkeeper who plays in the Swiss first league came to our lab for a two-hour motion capture session. During this session, we performed kicks with a real ball to mimic penalty kicks, and the goalkeeper performed dives and saves, executing all movements usually performed by the goalkeeper on the pitch when trying to stop a penalty kick. The goalkeeper was equipped with 49 infrared reflective markers, and his movements were captured using 12 infrared cameras (OptiTrack system, NaturalPoint, Inc.) at a sampling rate of 120 Hz. The skeleton and its movements were 'reconstructed' by the Motive software (NaturalPoint, Inc.). This reconstruction consisted in creating a skeleton consistent with the morphology of the physical

goalkeeper and in updating the skeleton rotations based on the 3D displacements of the markers over time. A 3D mesh of the avatar based on the morphological dimensions of the physical goalkeeper was then created with Fuse (Adobe). Overall, the whole process was very similar to that used to create and animate characters for video games or movies like Avatar.

Parameters of the Experiment

The time at which the dive was triggered could change from trial to trial based on two factors, namely the estimated time before foot-ball contact and the estimated level of performance of the player at this stage of the training. Before detailing how these parameters were estimated, we will define the three parameters which characterized each trial:

The parameter SIDE corresponded to the initial direction of the kick, with two possible values, namely SIDE $=$ **L** (left) or SIDE $=$ **R** (right). This parameter was the only one communicated to the player before each trial. The second parameter, KICK, corresponded to the necessity (or not) for the player to redirect the kick, noted KICK $=$ **RE** (redirection required) or KICK $=$ **NO** (no redirection required). This second parameter depended on both the initial target position and the side of the dive of the holographic goalkeeper. The third parameter was DEL, which corresponded to the delay/lag between the onset of the dive of the holographic goalkeeper and foot-ball contact (i.e., kick of the player). Note that the time before foot-ball contact could obviously not be enforced during the experiment. Instead, the run-up of each player was modeled using his previous run-ups, and for each trial, the time to foot-ball contact was estimated using the modeled run-up. For each player, the run-up was modeled with a time/radius mapping, i.e., by associating the time to foot-ball contact to the distance to the penalty mark

(sampling from 0 cm to 200 cm, using 1 cm steps). When the player made a run-up to take a penalty kick, SimulKick used the previously modeled run-ups to estimate in real-time the remaining run-up time and trigger the goalkeeper's dive as close as possible to the predicted time.

After each kick, we recorded the actual time interval (TIME) between the dive of the goalkeeper and the kick, as well as the result (RES) of the kick. The value of this latter parameter could be either **F** (when the ball missed the goal), **W** (when the ball was kicked towards the wrong side of the goal, namely the one where the goalkeeper dove), or **G** (when the ball was kicked towards the correct side of the goal, namely the open one). When RES $= \mathbf{F}$ or RES $= \mathbf{W}$ (see also player anticipation below), or when the difference between TIME and DEL was too large (see below), the kick was considered incorrect and was therefore repeated later in the session.

Measured Time and Predicted Delay. The predicted delay DEL and the actually recorded time TIME were never identical. Specifically, our algorithm predicted the time to foot-ball contact, but small, ecological variations of the player's run-up obviously affected this prediction. We modeled this phenomenon by assuming that :

$$\begin{aligned} \text{TIME} &= \text{DEL} + \epsilon \\ \epsilon &\sim \mathcal{N}(0, \varepsilon^2) \end{aligned} \quad (1)$$

where ϵ is a centered Gaussian random variable of variance ε^2, which encodes these small variations. This had two consequences on our model. First, we used the observed time TIME instead of DEL to assess the player's performance, as this is the value that was actually tested in the experiment. Second, we only used trials in which the difference was small enough, namely two standard deviations of the

Gaussian random variable, defined as 2ε. For larger variations, we assumed that the run-up of the player had an unusual pattern (for instance, the player stopped and waited for the goalkeeper to jump), making this trial significantly different in nature from the other trials. Such 'aberrant' trials were set aside during the analysis, and another occurrence of this very trial (i.e., with the same parameters) was repeated at a random point later during the session.

The other type of trial that was considered invalid was the anticipated redirection, which occurred when KICK $= \mathbf{NO}$ but RES $= \mathbf{W}$. In this case, the player was supposed to kick the ball towards the initially indicated target/side, but instead redirected the kick, and this even though it was not required because the goalkeeper actually dove to the opposite side of the goal. This behavior is antithetical to the main objective of the experiment, which was designed to test and train the players' ability to redirect the kick as late as possible in the run-up to the ball. Therefore, these trials were considered invalid and repeated at a random later point in the session. Also, the proportion of trials with anticipated redirection was used to adjust the level of performance of the player with KICK $= \mathbf{RE}$. This is because if the player anticipates the redirection, the task becomes menial and the results irrelevant. This proportion was used as a guess rate[44] in our model (see below).

Player Performance and Model

In order to provide personalized training parameters and to measure the evolution of a player's performance, our learning algorithm builds an internal representation of the performance of each player.

To this end, the kicks were split in four categories, depending on the SIDE and KICK values, i.e., the initial target side and whether the kick had to be redirected

or not.

The assessment of the result as well as the modeling of the player's performance were conducted independently for each side ($\text{SIDE} = \mathbf{L}$ and $\text{SIDE} = \mathbf{R}$). Therefore, and for the sake of brevity, SIDE is omitted in the following.

Modeling kicks for which no redirection was required. Supplemental Figure 4 depicts the Bayesian Network used to model the performance for non-redirected kicks (i.e., no redirection required). We modeled the probability of outcome of a kick without redirection ($\text{KICK} = \mathbf{NO}$) as follows:

$$\mathbb{P}(\text{RES} = \mathbf{F}|\text{KICK} = \mathbf{NO}) = p_N^{\mathbf{F},\mathbf{NO}}$$

$$\mathbb{P}(\text{RES} = \mathbf{W}|\text{KICK} = \mathbf{NO}) = (1 - \underbrace{\mathbb{P}(\text{RES} = \mathbf{F}|\text{KICK} = \mathbf{NO})}_{\text{Failure } \mathbf{NO}}) \times p_N^{\mathbf{RE}}$$

$$\mathbb{P}(\text{RES} = \mathbf{G}|\text{KICK} = \mathbf{NO}) = (1 - \underbrace{\mathbb{P}(\text{RES} = \mathbf{W}|\text{KICK} = \mathbf{NO})}_{\text{Anticipation}}) \times (1 - \underbrace{\mathbb{P}(\text{RES} = \mathbf{F}|\text{KICK} = \mathbf{NO})}_{\text{Failure } \mathbf{NO}})$$

where $p_N^{\mathbf{F},\mathbf{NO}}$ and $p_N^{\mathbf{RE}}$ are two unknown parameters that are assumed to be constant during a session N. The network first evaluates if the player missed the kick, with $\mathcal{B}(p_N^{\mathbf{F},\mathbf{NO}})$, a Bernoulli random variable of mean $p_N^{\mathbf{F},\mathbf{NO}}$. If the player did not miss the kick, the network then evaluates if the player anticipated the (re)direction of the kick, with an independent Bernoulli random variable of mean $p_N^{\mathbf{RE}}$. The kick was considered valid if the player neither missed nor anticipated.

Importantly, our model assumes that the probability of these events is independent from TIME and DEL. Specifically, when $\text{KICK} = \mathbf{NO}$, the player is not supposed to redirect the kick after the goalkeeper dive. Therefore, the task is not harder (resp. easier) for shorter DEL. Moreover, kicking the ball from the penalty mark to a target area that encompasses a third of the goal is a relatively easy task for professional football players. We therefore assumed that $\mathbb{P}(\text{RES} = \mathbf{F}|\text{KICK} = \mathbf{NO})$ should be relatively small, and that all kicks directed

to the wrong side of the goal result from an anticipated redirection of the penalty taker.

Modeling kicks requiring redirection. Supplemental Figure 5 depicts the Bayesian Network used to model the performance for redirected kicks (i.e., redirection required). The probability of scoring when a kick redirection is required ($\text{KICK} = \mathbf{RE}$) is assumed to depend on the time between the goalkeeper dive and foot-ball contact. This is modeled by:

$$\mathbb{P}(\text{RES} = \mathbf{G} | \text{KICK} = \mathbf{RE}) = (1 - \underbrace{p_N^{\mathbf{F},\mathbf{RE}}}_{\text{Failure } \mathbf{RE}}) \times \Big(\underbrace{p_N^{\mathbf{RE}}}_{\text{Anticipation}} + (1 - p_N^{\mathbf{RE}}) \underbrace{\Phi_{\mu_N, \sigma_N}(T_N)}_{\text{Redirection}} \Big)$$

$$\mathbb{P}(\text{RES} = \mathbf{F} | \text{KICK} = \mathbf{RE}) = 1 - \mathbb{P}(\text{RES} = \mathbf{G} | \text{KICK} = \mathbf{RE})$$

where $p_N^{\mathbf{F},\mathbf{RE}}$, $p_N^{\mathbf{RE}}$, μ_N, σ_N are four unknown parameters that are assumed to be constant during a session N, while T_N is the TIME value observed for this trial.

In other words, the model works as follows: First, it evaluates whether the player missed the kick, with $\mathcal{B}(p_N^{\mathbf{F},\mathbf{RE}})$, a Bernoulli random variable of mean $p_N^{\mathbf{F},\mathbf{RE}}$. If the kick was not missed, the network then evaluates if the player anticipated redirection, with an independent Bernoulli random variable of mean $p_N^{\mathbf{RE}}$. If the player anticipated redirection, then the kick likely succeeded, because in this case, redirection was planned, which means that no 'late' reprogramming was required. In this scenario, the task was much easier, especially for professional football players. If the player did not anticipate redirection, then the model assesses if the player was able to redirect the kick at the last moment. This probability is equals to $\Phi_{\mu_N, \sigma_N}(T_N)$, i.e., the value of the cumulative distribution function of a Gaussian random variable of mean μ_N and variance σ_N^2, evaluated on time T_N. The choice of this function is discussed below. If the player failed to redirect the kick, then the kick failed ($\text{RES} = \mathbf{F}$).

The Redirection Function.. In our model, the choice of the cumulative distribution function

$$\Phi_{\mu_N,\sigma_N}(T_N) = \frac{1}{\sqrt{2\pi\sigma_N^2}} \int_{t=-\infty}^{T_N} \exp\left(-\frac{(t-\mu_N)^2}{2\sigma_N^2}\right) dt \qquad (2)$$

for the redirection probability highlights the link between this particular problem and psychometric functions. In particular, the redirection task satisfies similar assumptions: it is a continuous, non-decreasing function of time (i.e., the longer the delay, the easier the redirection). In line with this remark, the method used to estimate the parameters of Φ has some similarity to Bayesian optimization techniques commonly used in psychophysics[44]. Similarly, the anticipation and failure rates $p_N^{\mathbf{F},\mathbf{RE}}$, $p_N^{\mathbf{F},\mathbf{NO}}$ and $p_N^{\mathbf{RE}}$ can be seen as variations of the guess and lapse rates, respectively. However, our setting yields a key difference : the intensity of the stimulus (here, the value of the delay) cannot be specified, due to the difference between TIME and DEL discussed before. Therefore, sampling methods such as maximum information[58] cannot be used directly here.

The Model Parameters.. As mentioned before, both directions of initial intent ($\mathbf{L}$ and $\mathbf{R}$) are treated independently in our model. Therefore, each parameter of the model has two values, which are computed on the two different datasets (Left kicks and Right kicks) using the same method. In our model, we used distinct failure rates for KICK $= \mathbf{NO}$ and $\mathbf{RE}$, ($p_N^{\mathbf{F},\mathbf{RE}}$ and $p_N^{\mathbf{F},\mathbf{NO}}$). This is because the two tasks are distinct, and redirecting a kick is harder than not redirecting it. Therefore, it is reasonable to assume that redirected kicks will miss the target more frequently than non-redirected kicks, and thus ($p_N^{\mathbf{F},\mathbf{RE}} > p_N^{\mathbf{F},\mathbf{NO}}$). The anticipation rate $p_N^{\mathbf{RE}}$ was assumed to be independent of the variable KICK because it encodes the intent of the player to redirect the ball before knowing whether it will be required or not (i.e., before observing KICK). Finally, μ_N, σ_N encode the

model inner representation of the player's performance in redirecting the kick. It is easy to see from (2) that μ_N represents the delay at which the player has a 50 $\%$ chance of successfully redirecting the kick, while σ_N quantifies how fast the player's performance improves (or worsens) when the delay varies. All parameters are supposed to stay constant within a session, but they might vary between sessions.

Adaptive Sampling

At the beginning of each session, the cumulative distribution function Ψ_N of the current belief about the values of μ_N is produced (see below Evolution of performance). Using Ψ_N, we set DEL_{min} and DEL_{max} as the values of μ that correspond to a 5% and 95% probability, respectively.

$$\begin{aligned} \text{DEL}_{\text{min}} &= \Psi_N^{-1}(0.05) \\ \text{DEL}_{\text{max}} &= \Psi_N^{-1}(0.95) \end{aligned} \tag{3}$$

Intuitively, DEL_{min} (resp. DEL_{max}) represents the lowest (resp. highest) likely delay for which the player may have a 50% chance of successfully redirecting his kicks. Then, the algorithm proceeds to sample 12 equidistant values of DEL that span the $[\text{DEL}_{\text{min}}, \text{DEL}_{\text{max}}]$ interval.

Updating the model

After each session, the different parameters of the model are estimated as follows: First, using the non-redirected kicks, $p_N^{\mathbf{F},\mathbf{NO}}$ and $p_N^{\mathbf{RE}}$ are estimated using the empirical averages. Then, using the redirected kicks, a posterior for $p_N^{\mathbf{F},\mathbf{RE}}, \mu_{N+1}, \sigma_{N+1}$ is computed for usage in the next session. Finally, an estimator of the current performance of the player is computed based on previous

performance and using a Kalman filter. This estimator is then returned to the experimenter as an indicator. Importantly, the estimator is only used for indicative feedback, but neither for the update of the player's performance model, nor for the data analysis. Supplemental Figure 6 depicts the sampling and updating steps.

Linear mixed-effects models

The two main factors were entered into the model as fixed effects, whereas the intercepts for the participants were entered as random effects. Four models were fitted, namely, 1. A model only including the intercept, 2. A model including the intercept and the Session as predictor, 3. A model including the intercept and both the Session and Redirection as predictors, and 4. A model including the intercept, the two factors as predictors and an interaction term. The four models were compared and p-values were obtained using likelihood ratio tests. The degrees of freedom were approximated using the Kenward-Roger method[59]. For each analysis, we also fitted three models including random slopes. The pattern of results was exactly the same whether the models included random slopes or not. However, the Akaike and Bayesian Information Criteria (i.e., AIC and BIC) were higher for the models including random slopes. We therefore do not report the results obtained with these 'overfitted' models.

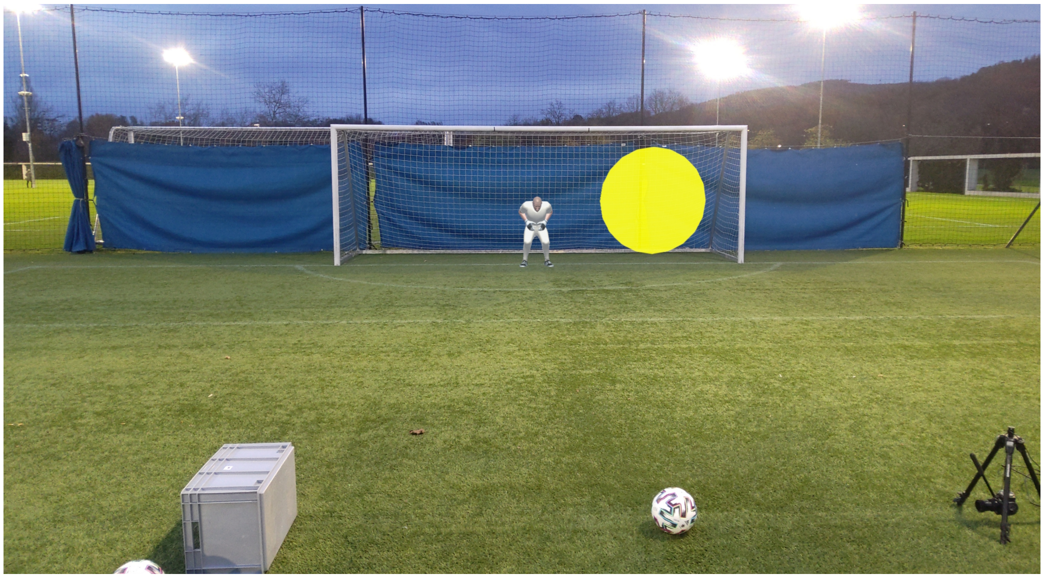

Supplemental Figure 1: Screenshot of a (right-footed) player's view just before starting the run-up to the ball. The holographic goalkeeper and the target area are displayed in the Microsoft HoloLens 2 Augmented-Reality headset. For this trial, the player is instructed to kick the ball to the left side of the goal, i.e., towards the yellow target area. However, if the goalkeeper dives to that side during the run-up, the player should try to redirect the kick to the right (i.e., open) side of the goal.

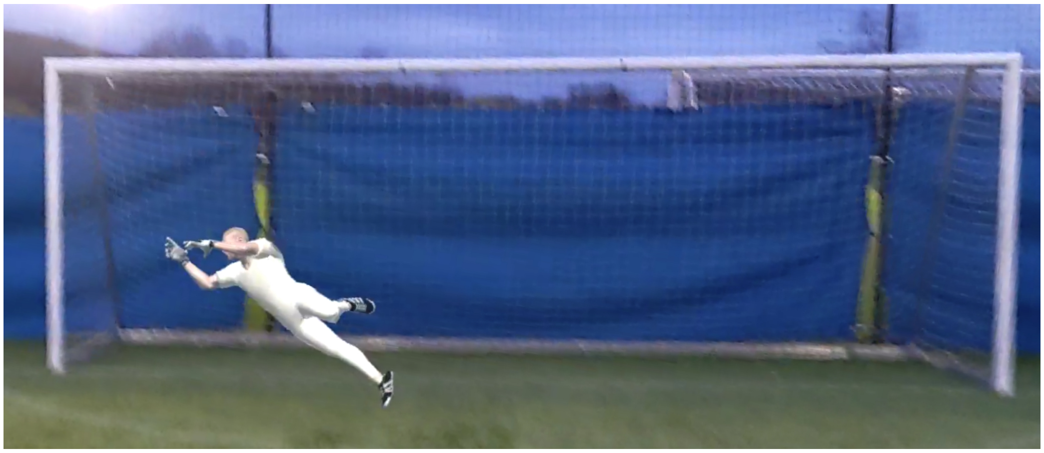

Supplemental Figure 2: The holographic goalkeeper is diving during the run-up of the player to the ball.

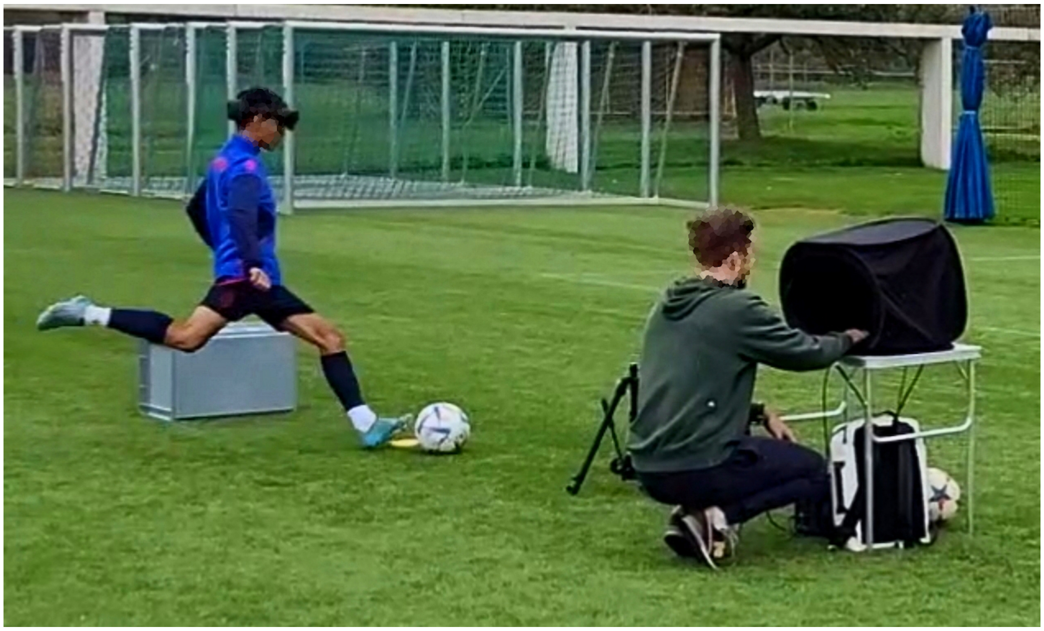

Supplemental Figure 3: A (right-footed) player about to kick the ball at the end of the run-up. The player sees the scene through the Microsoft Hololens 2 headset.

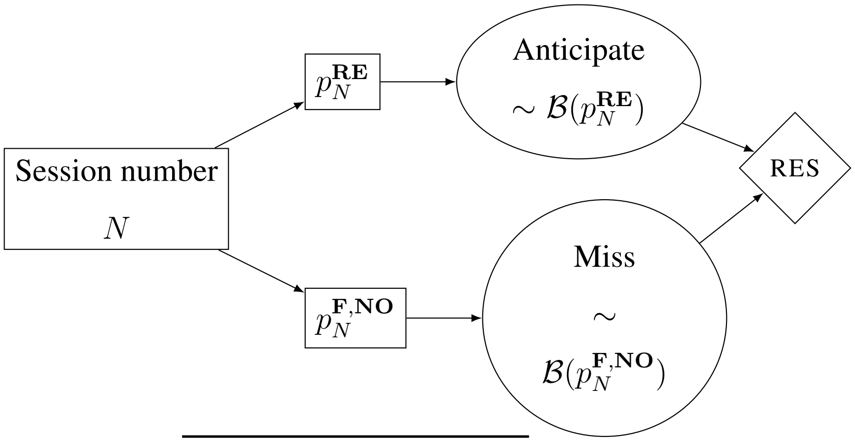

Miss	Anticipate	RES
True	-	**F**
False	True	**W**
False	False	**G**

Supplemental Figure 4: Bayesian Network used for modeling kicks for which no redirection was required. Rectangles represent parameters, ellipses random variables and diamonds the observed variables. Each random variable follows a Bernoulli distribution $\mathcal{B}$, and the relation between the hidden variables and the observed result is summarized in the joint Table.

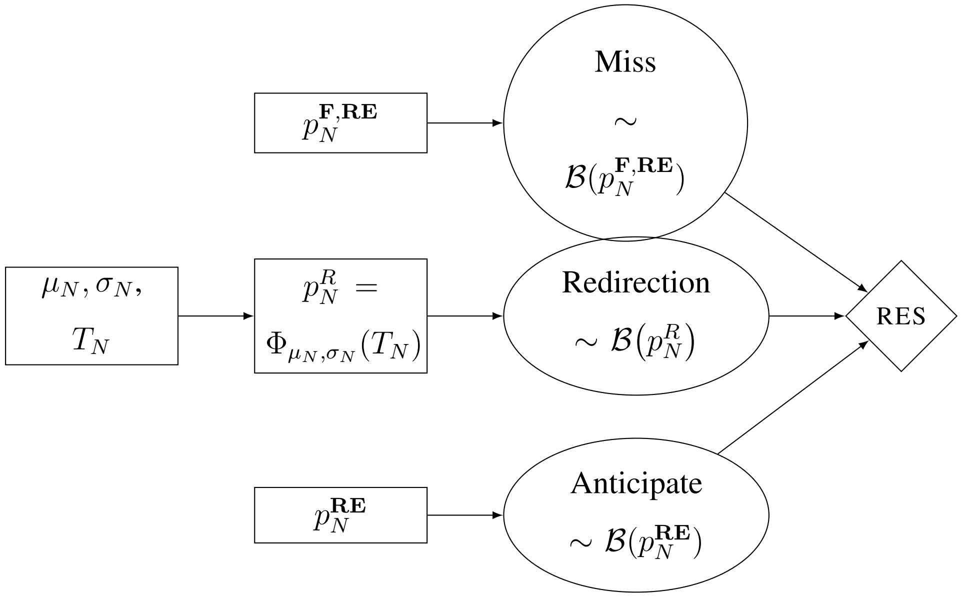

Miss	Anticipate	Redirection	RES
True	-	-	**F**
False	True	-	**G**
False	False	True	**G**
False	False	False	**F**

Supplemental Figure 5: Bayesian Network used for modeling the kicks requiring redirection. The session number N is also a parameter, but it has been removed to improve readability. Rectangles represent parameters, ellipses random variables, and diamonds the observed variables. Each random variable follows a Bernoulli distribution $\mathcal{B}$, and the relation between the hidden variables and the observed result is summarized in the joint Table.

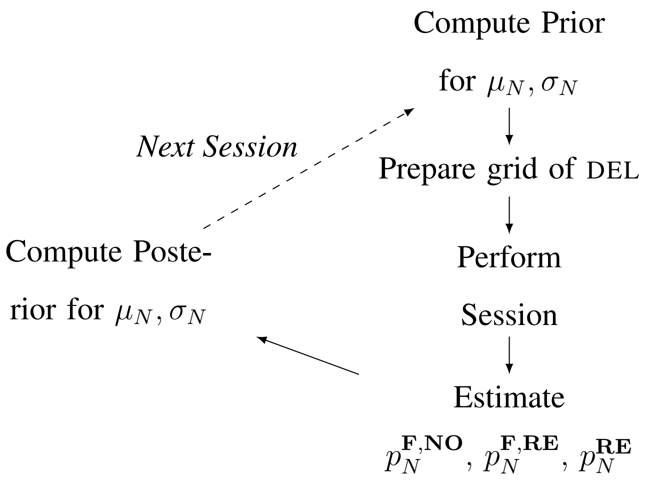

Supplemental Figure 6: Summary of the sampling and update of the model.
