# Supplementary material for: It’s not all in your feet: Improving penalty kick performance with human-avatar interaction and machine learning
Source: Innovation (Camb). 2024 Feb 6;5(2):100584. doi: 10.1016/j.xinn.2024.100584 (PMC10912701; doi:10.1016/j.xinn.2024.100584)
Supplement: Document S2. Article plus supplemental information [file mmc2.pdf]

www.the-innovation.org

It's not all in your feet: Improving penalty kick performance with human-avatar interaction and machine learning

Jean-Luc Bloechle,[1,4] Julien Audiffren,[1,4] Thibaut Le Naour,[2] Andrea Alli,[1] Dylan Simoni,[1] Gabriel Wüthrich,[3] and Jean-Pierre Bresciani[1,*]

*Correspondence: jean-pierre.bresciani@unifr.ch

Received: July 10, 2023; Accepted: January 24, 2024; Published Online: February 6, 2024; https://doi.org/10.1016/j.xinn.2024.100584

© 2024 This is an open access article under the CC BY license (http://creativecommons.org/licenses/by/4.0/).

GRAPHICAL ABSTRACT

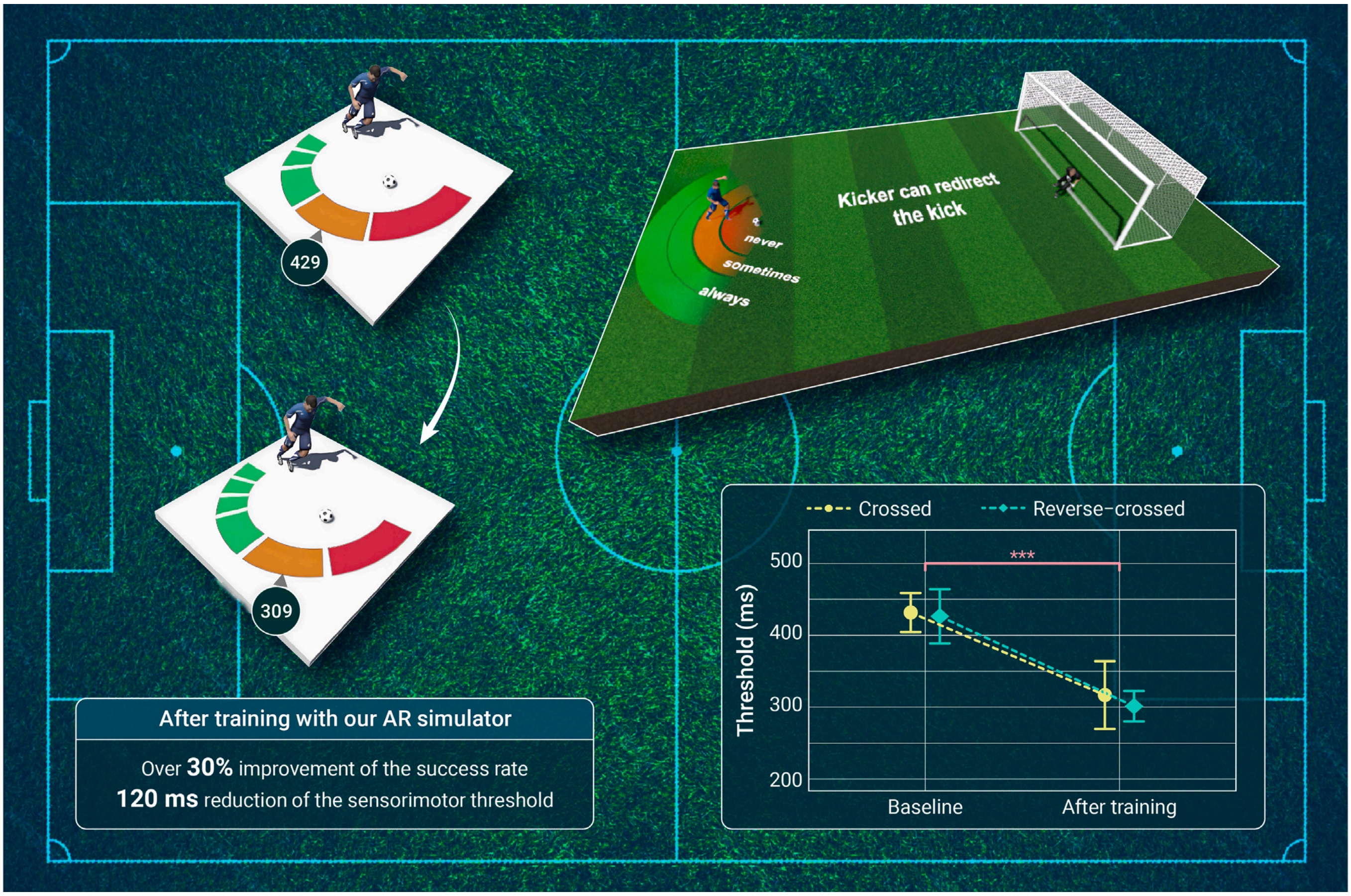

PUBLIC SUMMARY

- In major football competitions, over 20% of knockout games are decided by penalty shootouts.
- Thirty percent of the kicks are missed, notably because players lack specific and adapted training methods.
- We developed an augmented reality simulator with a holographic goalkeeper to train sensorimotor kicking skills.
- Ten sessions of machine learning-optimized training improved the sensorimotor skills of players by 28%.
- This translates into a 35% increase of the success rate, thereby constituting a powerful training tool.

It's not all in your feet: Improving penalty kick performance with human-avatar interaction and machine learning

Jean-Luc Bloechle,[1,4] Julien Audiffren,[1,4] Thibaut Le Naour,[2] Andrea Alli,[1] Dylan Simoni,[1] Gabriel Wüthrich,[3] and Jean-Pierre Bresciani[1,*]

[1]Control and Perception Laboratory, University of Fribourg, Bd Perolles 90, 1700 Fribourg, Switzerland

[2]Motion-up, Le Prisme, Place Albert Einstein, 56000 Vannes, France

[3]FC Basel 1893, Birsstrasse 320A, 4002 Basel, Switzerland

[4]These authors contributed equally

*Correspondence: jean-pierre.bresciani@unifr.ch

Received: July 10, 2023; Accepted: January 24, 2024; Published Online: February 6, 2024; https://doi.org/10.1016/j.xinn.2024.100584

© 2024 This is an open access article under the CC BY license (http://creativecommons.org/licenses/by/4.0/).

Citation: Bloechle J.-L., Audiffren J., Le Naour T., et al., (2024). It's not all in your feet: Improving penalty kick performance with human-avatar interaction and machine learning. The Innovation **5(2)**, 100584.

Penalty kicks are increasingly decisive in major international football competitions. Yet, over 30% of shootout kicks are missed. The outcome of the kick often relies on the ability of the penalty taker to exploit anticipatory movements of the goalkeeper to redirect the kick toward the open side of the goal. Unfortunately, this ability is difficult to train using classical methods. We used an augmented reality simulator displaying an holographic goalkeeper to test and train penalty kick performance with 13 young elite players. Machine learning algorithms were used to optimize the learning rate by maintaining an optimal level of training difficulty. Ten training sessions of 20 kicks reduced the redirection threshold by 120 ms, which constituted a 28% reduction with respect to the baseline threshold. Importantly, redirection threshold reduction was observed for all trained players, and all things being equal, it corresponded to an estimated 35% improvement of the success rate.

INTRODUCTION

In the last 40 years, penalty kicks have often been decisive in international football competitions.[1] In the knockout phases of the FIFA World Cup, 21% of the games were decided by penalty shootouts, as was the final of the recent 2022 World Cup. This number reaches 28% regarding UEFA Champions League finals. In shootout sessions, about 30% of the penalties are missed.[2,3] Such a high miss rate is surprising when considering the advantage of the player over the goalkeeper. Indeed, the goalkeeper can only cover a small portion of the 18 square meters goal area. In addition, the goalkeeper needs about 900 ms to dive and reach a side of the goal (ie, 200 ms of visual reaction time[4] and 700 ms of movement/diving time[5]), whereas the ball reaches the goal less than 500 ms after the kick.[6,7] If the player shoots to a side of the goal, the goalkeeper must start moving at least 300 ms before foot-ball contact to stand a chance to block the kick. And indeed, professional goalkeepers anticipate-dive to a side of the goal in about 95% of penalty kicks.[8] Consequently, penalty takers developed a strategy consisting in "awaiting" an early dive of the goalkeeper that would allow them to kick the ball to the "open" side of the goal.[3] Specifically, the player selects a side in advance, but this plan is subject to alterations depending on the goalkeeper's movements.[9–15] This strategy, called goalkeeper dependent, is adopted by 75% of professional penalty takers.[3] With this strategy, three scenarios are possible. If the goalkeeper does not move before foot-ball contact, the player kicks toward the initially selected side. If, before foot-ball contact, the goalkeeper dives to the side opposite the one selected by the player, the player also kicks toward the initially selected side. If, however, the goalkeeper dives (before foot-ball contact) to the side initially selected by the player, this latter must modify his/her motor plan during the run-up to kick the ball toward the open side of the goal. Unfortunately, redirecting the kick is not always possible. In particular, the sensorimotor loops underlying kick redirection require time to process visual information relative to the goalkeeper and to modify the initial motor plan. Put differently, successfully redirecting the kick to score the penalty is only possible if there is enough time left before foot-ball contact.

The minimum time needed to successfully modify an ongoing movement, the "new" movement accurately corresponding to the desired outcome, has been extensively studied. Most of these studies are based on arm reaching movements, and perturbations are introduced during the movement.[16–24] Visually driven corrections of such reaching movements are efficient, smooth, and occur with short latency. Deviations of the hand trajectory are usually observed between 280 and 350 ms after perturbation,[17,25,26] although under certain conditions, they can occur as early as 130 ms after perturbation.[18] Interestingly, when measured in comparable conditions and with similar tasks, online modifications occur almost twice faster as visual reaction times.[19] Therefore, many authors have suggested that as opposed to typical reaction times, online modifications are largely automatic[16–18,27] and could rely on subcortical control loops.[19] Unfortunately, in contrast with the abundant literature describing the characteristics and efficiency of the sensorimotor loops controlling simple reaching movements, little is known about the online control of more complex movements. This is notably because complex movements are harder to study in a controlled fashion.

Here, we quantified the minimum time necessary to successfully redirect a penalty kick, and more importantly, we assessed whether this time can be "shortened" using appropriate training. We modified the double-step paradigm traditionally used to study the online control of reaching movements, and adapted it to a realistic penalty kick simulation. Specifically, we developed an augmented reality simulator in which football players tried to score penalty kicks on a real soccer pitch, with a real soccer ball and a real goal, but facing a human-like holographic goalkeeper (see Figure 1). During the run-up to the ball, the holographic goalkeeper dove to one side of the goal as real goalkeepers anticipate-dive. For half of the kicks, the dive forced the penalty taker to redirect the kick (see Figure 2). For each player and each trial, the dive onset depended on the estimated time to foot-ball contact. This time was estimated using a time/radius mapping algorithm based on kinematic information relative to both the current and previous run-ups to the ball. Dive onset was adjusted from trial to trial as a function of two factors, namely (1) the actual time of the dive before foot-ball contact (as measured on previous trials) and (2) the associated performance of the penalty taker, ie, his sensorimotor ability to successfully redirect the kick when needed. As this sensorimotor ability improved, the task became more and more difficult. Put differently, as the minimum time required by the player to successfully redirect the kick decreased, the goalkeeper dive occurred later in the run-up to the ball, which made it harder for the player to redirect the kick. A Bayesian network was used to model the player's current level of performance and its evolution in order to adjust the difficulty of the task and optimize the training rate. This optimization aimed at bringing each player to the best possible performance in the minimum training time.

RESULTS

The first two sessions were used to estimate the baseline performance of each player, namely the 50% redirection threshold. On average, the baseline redirection threshold was 429.02 ms (±53.46, range: 319–536 ms). After 10 training sessions, the 50% redirection threshold dropped to 309.08 ms (±59.76, range: 213–488 ms). This 120 ms threshold reduction was significant ($\chi^2(1) = 44.15$, $p < 0.001$), it explained 54% of the variance (marginal $R^2 = 0.54$), and it constituted a 28% reduction as compared with baseline.

We then assessed whether and how the side of the required redirection affected the redirection threshold. When the player had to redirect the kick toward the side opposite the kicking foot (ie, redirection to the left for a right-footed player), redirection was defined as crossed redirection. When the player had to

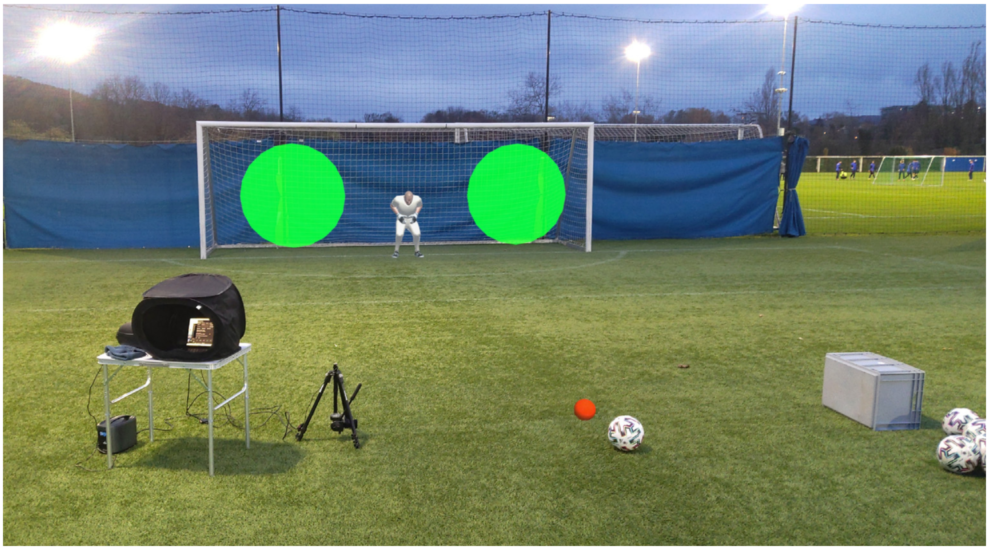

Figure 1. Visual scene viewed through a Microsoft HoloLens 2 headset The player's view after the ball has been positioned on the penalty mark. The red holographic sphere indicates the player's gaze location.

redirect the kick toward the side of the kicking foot (ie, redirection to the right for a right-footed player), redirection was defined as reverse-crossed redirection.[28] The redirection threshold was not affected by the redirection side ($\chi^2(1) = 0.61$, $p = 0.43$), and there was no interaction between the session and the redirection side ($\chi^2(1) = 0.15$, $p = 0.70$). The redirection side did not affect either the baseline threshold (431.62 ± 45.05 ms vs. 426.41 ± 62.52 ms, $p = 0.68$, Bayes factor = 0.29) or the training-evoked threshold reduction (−114.77 ± 78.72 ms vs. −125.10 ± 46.93 ms, $p = 0.79$, Bayes factor = 0.31). Importantly, as shown in Figure 3, a significant threshold reduction was observed after training for both crossed ($p < 0.01$, $R = 0.82$) and reverse-crossed redirection ($p < 0.001$, $R = 0.88$). Figure 4 shows the probability of reduction of the redirection threshold as a function of the reduction amplitude (in ms) for each of the players and the two redirection sides. This probability (derived by our Bayesian network) ranged from 0.29 to 0.99 (mean = 0.84 ± 0.17, median = 0.88), and all values but two were larger than 0.7, indicating a high probability of improvement. The probability of observing such an outcome by chance, namely 24 improvements out of 26 draws, is about 1/100,000 (as assessed by a two-tailed binomial test). We also computed the probability that the training-evoked improvement be larger than one standard deviation. This probability ranged from 0.16 to 0.94 (mean = 0.7 ± 0.23, median = 0.73), with all values but four above 0.5.

From an applied perspective, what coaches and football professionals probably want to know is how the redirection threshold reduction translates in terms of success rate. When taking the baseline redirection threshold as reference performance (ie, 429 ms), the training-evoked threshold reduction corresponds to an estimated 34% improvement of the success rate. The estimated success rate rises from 49% ± 21% and 52% ± 18% before training (for crossed and reverse-crossed redirection, respectively) to 89% ± 7% and 81% ± 21% after training. The before vs. after difference is significant in both cases ($p < 0.001$, $R = 0.88$ and $p < 0.01$, $R = 0.80$). When taking the redirection threshold measured after training as reference (ie, 309 ms), the average improvement is 36%, and the estimated success rate rises from 14% ± 12% and 15% ± 10% before training to 53% ± 14% and 47% ± 23% after training. Again, the before vs. after difference is significant in both cases ($p < 0.001$, $R = 0.88$ and $p < 0.01$, $R = 0.83$). Figure 5 shows the effect of training on the estimated probability of successful redirection as a function of time.

DISCUSSION

Only 10 sessions of 20 kicks with our simulator resulted in a 120 ms (ie, 28%) reduction of the redirection threshold. All things being equal, this threshold reduction translates into a sizable 35% improvement of the success rate. Importantly, the probability of the training to reduce the redirection threshold was 84% on average, and superior to 70% in 24 of the 26 player-side combinations. Similarly, the training substantially increased the probability of success rate for 23 of the 26 player-side combinations, and this over a large time range of anticipation-dive of the goalkeeper.

Very few studies previously attempted to address the control and redirection of penalty kicks.[29,30] In these studies, the approach to the problem was different from ours, and penalty simulations were non-realistic. Specifically, one study[29] measured choice reaction times (lever-tilting task), which are different from online responses.[19,23,31] The other study[30] used light bulbs instead of a goalkeeper, which can alter the player's behavior[32] and attention orienting processes.[33] In addition, both studies exclusively investigated redirection thresholds. As opposed to that, we combined augmented reality, human-avatar interaction and machine learning algorithms to develop an ecologically valid simulation allowing us to reduce the redirection threshold and improve success rate. Our simulator is used on a soccer pitch with soccer balls, and the visual stimulus triggering the redirection response is an holographic goalkeeper having the same size and moving exactly as a real goalkeeper, thereby matching realistically the real penalty kick situation. Those factors confer physical, biomechanical, and perceptive-cognitive

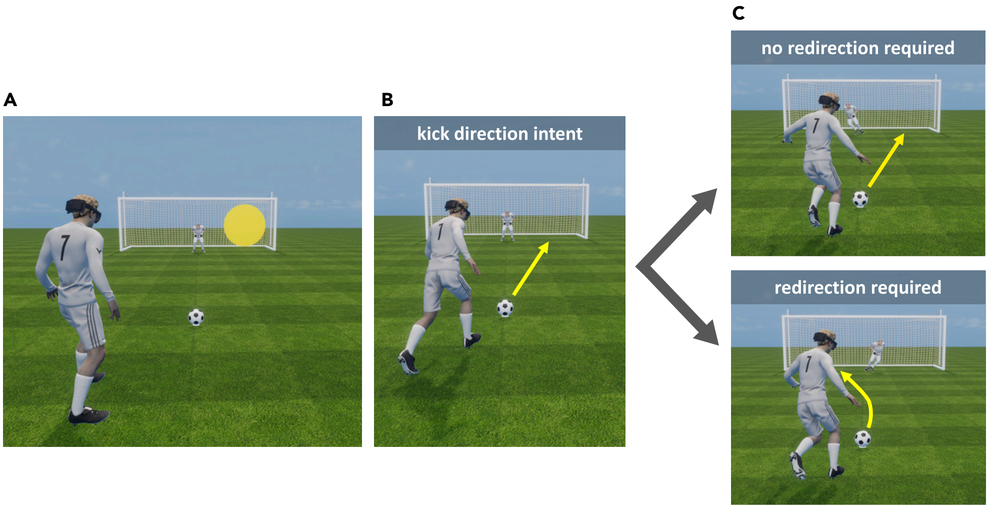

Figure 2. The different stages of a trial for the penalty taker (A) The player is about to start running up. The yellow target indicates where to kick the ball. (B) The player starts running up to the ball with the "objective" to kick the ball to the previously displayed target area. (C) Upper panel: no redirection trial—during the run-up, the holographic goalkeeper dives to the side opposite the "target side"; no kick redirection is required. Lower panel: redirection trial—the holographic goalkeeper dives to the target side; the penalty taker must change his motor plan and redirect the kick toward the open side of the goal.

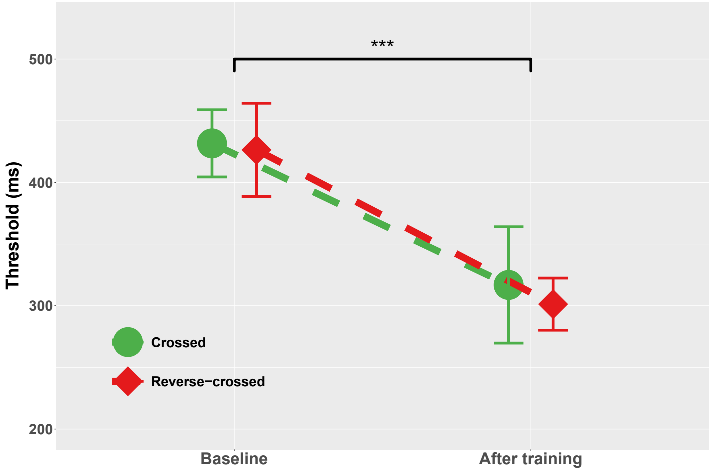

Figure 3. Average redirection threshold before (baseline) and after training The redirection threshold was significantly lower after training ($p < 0.001$). The pattern is similar for crossed (green, redirection toward the side opposite the kicking foot) and reverse-crossed redirection (red, redirection toward the side of the kicking foot). The error bars represent the 95% confidence interval.

fidelity to our simulator,[34–36] making the task at hand more engaging[37,38] and increasing transfer likelihood.[39–42] Finally, our Bayesian network grants a more accurate and reliable estimation of the minimum time required to successfully redirect the kick. Specifically, both anticipation behavior and the "global" rate of failed kicks (ie, the proportion of failed kicks that are not imputable to the redirection constraint) are taken into account to limit anticipation-related bias when estimating the threshold value.[43–45] Therefore, our Bayesian network allows us to finely model the individual performance of each player (see Figures S4 and S5), and the player model is continuously updated by integrating the performance on the "new" trials (see Figure S6).

As mentioned above, an important proportion of games in international football competitions are decided by penalty shootouts. These sessions have a "dramatic flavor," both for the teams and their supporters. Therefore, the fear of missing puts a lot of pressure and stress on the penalty taker, especially when he/she has a lot riding on his/her kick.[46–50] This psychological pressure has a negative impact on the success rate.[51] Being well prepared and more aware of your skills is probably one of the most efficient ways to cope with such stressful situations.[52,53] Accordingly, the ability to train players to successfully redirect the kick later in the run-up will not only increase their success rate, but will also contribute to reduce their stress.[54] In that respect, our simulator constitutes a unique training tool allowing players to practice penalty kicks and improve their sensorimotor skills in a way that would be impossible otherwise. In particular, the simulator precisely triggers the dive of the goalkeeper based on the run-up of the player, which would be impossible with a real goalkeeper. Coupled with our optimization algorithms, this grants the possibility to permanently adjust the difficulty of the training to keep the athlete in the "optimal challenge zone" (ie, neither too easy nor too difficult). This maintains the athlete at a high level of motivation and optimizes his/her learning pace.[55,56] In addition, the virtual goalkeeper can perform an infinite number of successive dives without risking any injury, which would be impossible with a real goalkeeper. Thus, our simulator provides an unparalleled tool to flexibly organize targeted training sessions.

As a final thought, we should mention that although the sensorimotor skills trained with our simulator seem very specific, they are not. Specifically, being able to redirect a penalty kick based on the visually detected movements of the goalkeeper is very similar to being able to redirect a pass based on the perceived movements of teammates and opponents. In that respect, we believe that the sensorimotor skills trained with our simulator would transfer, at least to some extent, to all game situations in which the player should pass/kick the ball under time constraint, eg, when pressed by a direct opponent or when about to pass the ball to a partner who is now marked or has changed position. Almost every time a player passes the ball, there is more than one passing option. The "best" option quickly changes because partners and opponents are constantly moving. Being able to redirect the kick shortly before kicking the ball increases the chances to deliver the ball to the best positioned partner at this very moment. This applies to all players on the pitch. Slight modifications to our simulator will grant the possibility to manipulate and control all relevant factors with precise timing to optimize progress rate. Therefore, the approach developed in this study should find larger applications, be it in football or other sports in which being able to modify the planned/ongoing action as late as possible provides a decisive advantage as, for instance, tennis or ice hockey.

MATERIALS AND METHODS

Participants

Thirteen young elite football players (U16 to U18 from FC Luzern and FC Basel) participated in the experiment (mean age = 15.77 ± 0.73; age range: 15–17; 13 male, 5 left-footed). Eight of them played for the U15, U16, and/or U17 Swiss national team. All were able-bodied with normal or corrected-to-normal vision. The study was performed in accordance with the ethical standards laid down in the 1964 Declaration of Helsinki and approved by the Ethics Committee of the University of Fribourg. Participants had the option to withdraw from the study at any time without penalty and without having to give a reason.

Setup/apparatus

The experiment was performed in the penalty area of a football pitch (grass), with a football goal and official match balls. The players were dressed in football outfits, and wore a Microsoft HoloLens 2 headset,[57] which is a "see-through" augmented reality headset. The headset was used to display the virtual part of the visual scene. When the luminosity was very high, a homemade filter (dark plastic film) was applied on the headset to increase the contrast of the virtual scene. The headset was also used to track the position of the player on the pitch. A LIDAR sensor (TeraRanger Evo 60m, sampling frequency of 240Hz, USB connection) fixed on a tripod was positioned to the side of the ball at a 1.5-m distance. The LIDAR was always positioned on the kicking foot side, and it was used to time and record foot-ball contact. A gray box opposite the LIDAR (2 m from the ball) reflected the laser beam after the kick. A laptop used to run the experiment and a mobile phone used as Wi-Fi hotspot (network communication between the laptop and the headset) were on a table next to the LIDAR. A portable PowerStation ensured the electric charging of all devices.

Virtual scene and holographic goalkeeper

The virtual scene was displayed in the headset. It consisted of the holographic goalkeeper and of colored 3D spheres and 2D target areas, depending on the phase of the trial (see procedure). The SimulKick application (see supplemental information) managed the scene and animated the holographic goalkeeper in real time, notably triggering his dives. The animations were based on the motion-captured movements of a professional goalkeeper (see supplemental information).

Procedure

At the beginning of each trial, the holographic goalkeeper was in the middle of the goal. The player had to put the ball on the penalty mark, which was highlighted by a red holographic sphere. This sphere turned green once the ball was on the mark. Two holographic target areas (2D disc, diameter 200 cm/10.4° of visual angle in diameter) were then displayed in red next to the left and right posts. Once the player was in his "starting" position for the run-up (at least 2 m from the ball), the two holographic targets turned green (see Figure 1). Before starting running up, the player had to fixate the goalkeeper's head for 3 s. One of the targets was then switched off and the other one turned yellow. The yellow target indicated where to kick the ball (left or right side of the goal, see Figure S1) and the player could initiate the run-up. The yellow target was switched off during the run-up, when the player was at a 2-m distance from the ball. During the run-up, the holographic goalkeeper dove to one side of the goal (left or right, see Figure S2). When the goalkeeper dove to the side opposite the previously displayed yellow target, no kick redirection was required. When the goalkeeper dove to the side where the yellow target was previously lit, the player had to redirect the kick toward the other side of the goal (opposite the initial target position). In other words, the player always had to kick the ball toward the "open side" of the goal. Figure 2 summarizes the kicking options for the player. The kick was successful when the player redirected the kick without anticipating redirection (see supplemental information). Figure S3 shows a player about to kick the ball. After the dive and the kick, the goalkeeper walked back to the center of the goal. The goalkeeper was displayed for the whole duration of the session, with different animations depending on the "stage" of the penalty kick (ie, before, during, or after the run-up). After each kick, the actual time of the goalkeeper dive and the success of the kick were registered in KickManager (see supplemental information). Each training session consisted of 20 penalty kicks, for a total duration of 15 min per session. Within any given session, the initial target side was always the same (right or left). Out of the 20 kicks, 12 randomly selected kicks required redirection (60% of the kicks). Each player performed

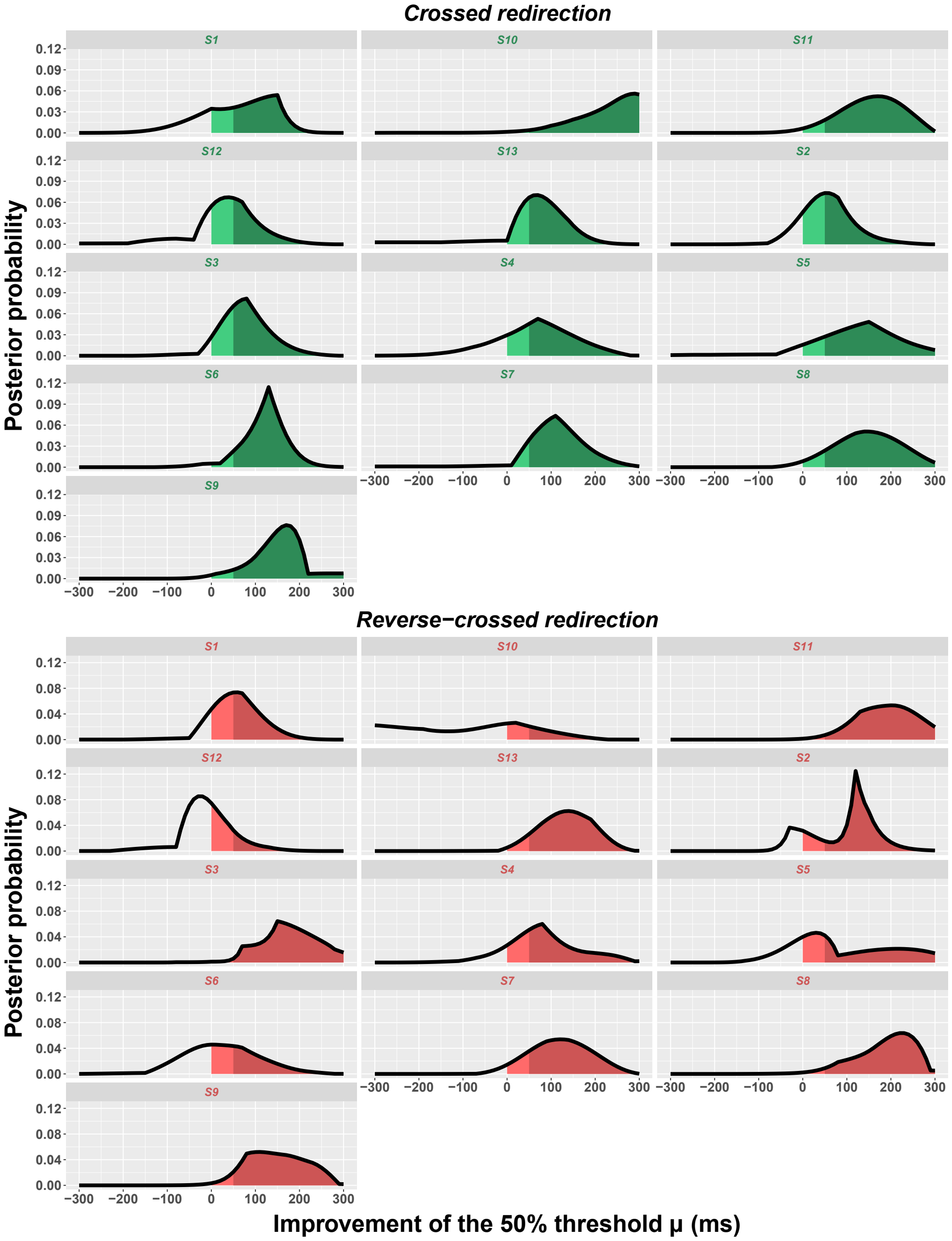

Figure 4. Probability of improvement of the redirection threshold for crossed (green) and reverse-crossed (red) redirection For each player (*Si*), the curve represents the estimated distribution of the redirection threshold after training (relative to baseline performance). The area under the curve for X values larger than 0 (i.e., green-shaded area for crossed redirection and red-shaded area for reverse-crossed redirection) shows the probability of improvement of the redirection threshold, i.e., the probability that the redirection threshold be lower after training. The dark-shaded area corresponds to the probability that the improvement be larger than one standard deviation.

two to three training sessions per week, and there was never more than one training session per day.

Triggering of the goalkeeper dive

The goalkeeper dive was triggered by SimulKick during the player's run-up. The onset of the dive could change from trial to trial based on (1) the estimated time before foot-ball contact and (2) the estimated level of performance of the player at this stage of the training (see supplemental information).

Data analysis

The session (baseline vs. after training) and the direction (crossed vs. reverse-crossed) of the required redirection were within subject factors (repeated measures). The dependent variable was the 50% redirection threshold, namely the minimum time required to successfully redirect the kick 50% of the time. The effect of the two factors and their interaction on the dependent variable was assessed using a linear mixed-effects modeling approach (see supplemental information). For each factor, the effect size was computed using the marginal R^2. Direct comparisons between means were performed using Wilcoxon signed-rank tests for repeated measures, and the effect size was computed using Pearson's R. When the Wilcoxon test was non-significant, we additionally computed the Bayes factor.

REFERENCES

1. Dalton, K., Guillon, M., and Naroo, S.A. (2015). An analysis of penalty kicks in elite football post 1997. Int. J. Sports Sci. Coach. ***10***: 815–827.
2. Franks, I., and Harvey, T. (1997). Cues for goalkeepers: Hightech methods used to measure penalty shot response. Soccer Journal ***42***: 30–38.
3. Kuhn, W. (1988). In in Science and Football, T. Reilly, A. Lees, and K. Davids, et al., eds., pp. 489–492.
4. Schmidt, R.A., Lee, T.D., Winstein, C., et al. (2018). Motor Control and Learning: A Behavioral Emphasis (Human Kinetics).
5. Kerwin, D.G., and Bray, K. (2010). The Engineering of Sport 6. In Volume 1: Developments for Sports (Springer), pp. 321–326.
6. Miller, C. (1998). He Always Puts it to the Right: A History of the Penalty Kick (Orion).
7. Palacios-Huerta, I. (2003). Professionals play minimax. Rev. Econ. Stud. ***70***: 395–415.
8. Bar-Eli, M., Azar, O.H., Ritov, I., et al. (2007). Action bias among elite soccer goalkeepers: The case of penalty kicks. Journal of Economic Psychology ***28***: 606–621.
9. Cook, R., Bird, G., Lünser, G., et al. (2012). Automatic imitation in a strategic context: players of rock-paper-scissors imitate opponents' gestures. Proc. Biol. Sci. ***279***: 780–786.
10. Belot, M., Crawford, V.P., and Heyes, C. (2013). Players of Matching Pennies automatically imitate opponents' gestures against strong incentives. Proc. Natl. Acad. Sci. USA ***110***: 2763–2768.
11. Naber, M., Vaziri Pashkam, M., and Nakayama, K. (2013). Unintended imitation affects success in a competitive game. Proc. Natl. Acad. Sci. USA ***110***: 20046–20050.

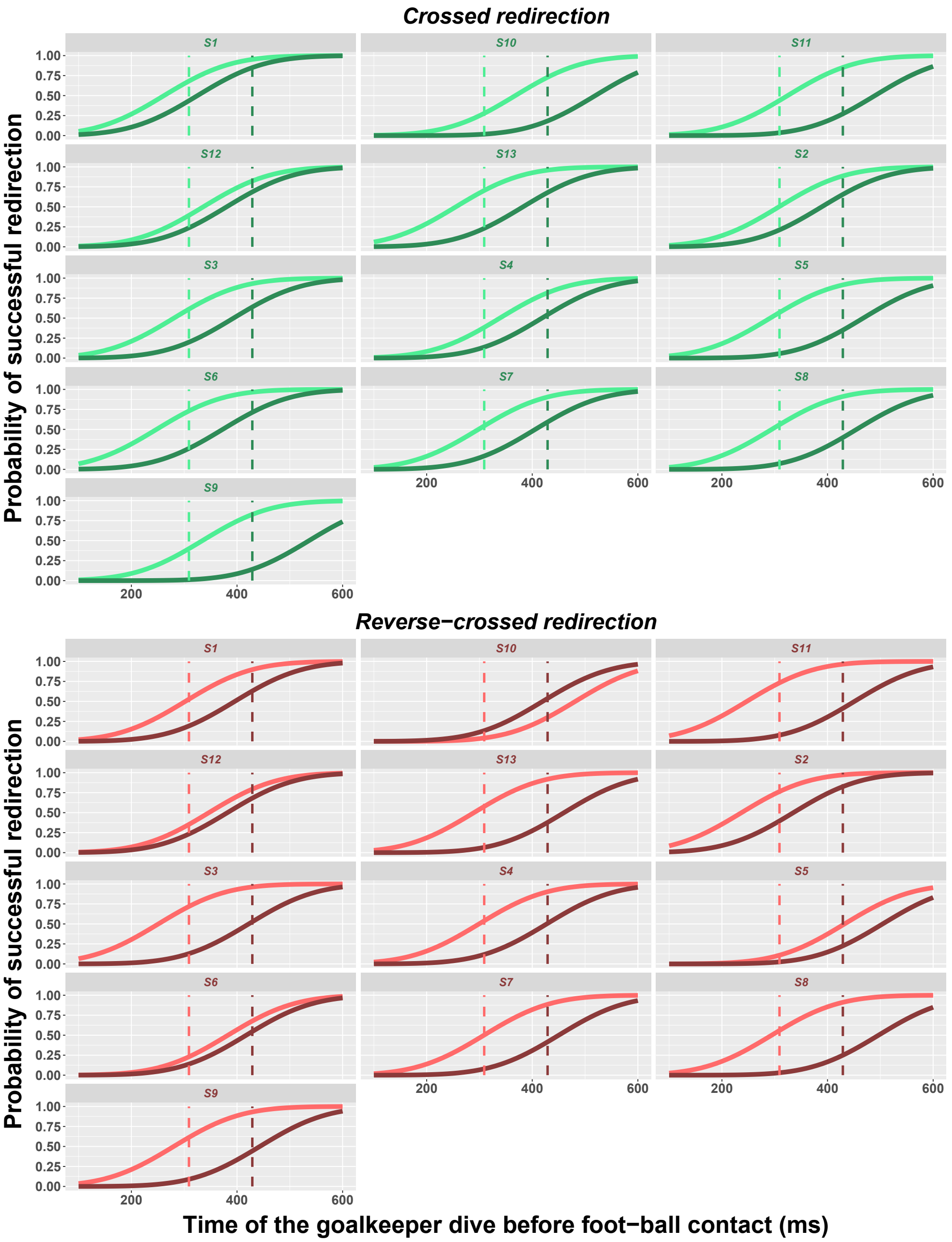

Figure 5. Probability of successful redirection for crossed (green) and reverse-crossed (red) redirection The curves represent the estimated probability of successfully redirecting the kick as a function of the time of the goalkeeper dive before foot-ball contact. For each player (S*i*), the dark-colored curve shows the estimated baseline probability, whereas the light-colored curve shows the estimated probability after training. For any X value, the Y value difference between the two curves corresponds to the training-evoked change of probability. The light-colored curve is almost always above the dark-colored curve, indicating an increase of the probability to successfully redirect the kick after training. The dashed vertical lines indicate the average (population-wise) redirection threshold before (dark colored) and after training (light colored).

12. Era, V., Aglioti, S.M., Mancusi, C., et al. (2020). Visuo-motor interference with a virtual partner is equally present in cooperative and competitive interactions. Psychol. Res. ***84***: 810–822.
13. Boukarras, S., Özkan, D.G., Era, V., et al. (2022). Midfrontal theta transcranial alternating current stimulation facilitates motor coordination in dyadic human-avatar interactions. J. Cogn. Neurosci. ***34***: 897–915.
14. Sacheli, L.M., Musco, M.A., Zazzera, E., et al. (2022). How shared goals shape action monitoring. Cereb. Cortex ***32***: 4934–4951.
15. Moreau, Q., Tieri, G., Era, V., et al. (2022). The performance monitoring system is attuned to others' actions during dyadic motor interactions. Cereb. Cortex ***33***: 222–234.
16. Goodale, M.A., Pelisson, D., and Prablanc, C. (1986). Large adjustments in visually guided reaching do not depend on vision of the hand or perception of target displacement. Nature ***320***: 748–750. ISSN: 0028-0836 (Print) 0028-0836 (Linking).
17. Prablanc, C., and Martin, O. (1992). Automatic control during hand reaching at undetected two-dimensional target displacements. J. Neurophysiol. ***67***: 455–469. 0022-3077 (Print) 0022-3077 (Linking).
18. Day, B.L., and Lyon, I.N. (2000). Voluntary modification of automatic arm movements evoked by motion of a visual target. Exp. Brain Res. ***130***: 159–168. ISSN: 0014-4819 (Print) 0014-4819 (Linking).
19. Day, B.L., and Brown, P. (2001). Evidence for subcortical involvement in the visual control of human reaching. Brain. ***124***: 1832–1840. ISSN: 0006-8950 (Print) 0006-8950 (Linking).
20. Gritsenko, V., Yakovenko, S., and Kalaska, J.F. (2009). Integration of predictive feedforward and sensory feedback signals for online control of visually guided movement. J. Neurophysiol. ***102***: 914–930. 0022-3077 (Print) 0022-3077 (Linking).
21. Gritsenko, V., and Kalaska, J.F. (2010). Rapid online correction is selectively suppressed during movement with a visuomotor transformation. J. Neurophysiol. ***104***: 3084–3104. ISSN: 1522-1598 (Electronic) 0022-3077 (Linking).
22. Wijdenes, L.O., Brenner, E., and Smeets, J.B.J. (2013). Comparing online adjustments to distance and direction in fast pointing movements. J. Mot. Behav. ***45***: 395–404. ISSN: 1940-1027 (Electronic) 0022-2895 (Linking).
23. Reichenbach, A., Thielscher, A., Peer, A., et al. (2009). Seeing the hand while reaching speeds up on-line responses to a sudden change in target position. J. Physiol. ***587***: 4605–4616. ISSN: 1469-7793 (Electronic) 0022-3751 (Linking).
24. Sarlegna, F.R., and Mutha, P.K. (2015). The influence of visual target information on the online control of movements. Vision Res. ***110***: 144–154. ISSN: 1878-5646 (Electronic) 0042-6989 (Linking).
25. Johnson, H., Van Beers, R.J., and Haggard, P. (2002). Action and awareness in pointing tasks. Exp. Brain Res. ***146***: 451–459. ISSN: 0014-4819 (Print) 0014- 4819 (Linking).
26. Sarlegna, F., Blouin, J., Bresciani, J.-P., et al. (2003). Target and hand position information in the online control of goal-directed arm movements. Exp. Brain Res. ***151***: 524–535. ISSN: 0014-4819 (Print) 0014-4819 (Linking).
27. Pisella, L., Gréa, H., Tilikete, C., et al. (2000). An 'automatic pilot' for the hand in human posterior parietal cortex: toward reinterpreting optic ataxia. Nat. Neurosci. ***3***: 729–736.

28. Nagasawa, Y., Demura, S., Matsuda, S., et al. (2011). Effect of Differences in Kicking Legs, Kick Directions, and Kick Skill on Kicking Accuracy in Soccer Players. Journal of Quantitative Analysis in Sports ***7***: 9.
29. Morya, E., Ranvaud, R., and Pinheiro, W.M. (2003). Dynamics of visual feedback in a laboratory simulation of a penalty kick. J. Sports Sci. ***21***: 87–95.
30. Van der Kamp, J. (2006). A field simulation study of the effectiveness of penalty kick strategies in soccer: late alterations of kick direction increase errors and reduce accuracy. J. Sports Sci. ***24***: 467–477.
31. Le Naour, T., Papinutto, M., Lobier, M., et al. (2023). Controlling the trajectory of a moving object substantially shortens the latency of motor responses to visual stimuli. iScience ***26***: 106838.
32. Navarro, M., van der Kamp, J., Ranvaud, R., et al. (2013). The mere presence of a goalkeeper affects the accuracy of penalty kicks. J. Sports Sci. ***31***: 921–929. ISSN: 1466-447X (Electronic) 0264-0414 (Linking).
33. Posner, M.I. (1980). Orienting of attention. Q. J. Exp. Psychol. ***32***: 3–25.
34. Gray, R. (2019). Anticipation and Decision Making in Sport 342-358 (Routledge).
35. Harris, D.J., Bird, J.M., Smart, P.A., et al. (2020). A framework for the testing and validation of simulated environments in experimentation and training. Front. Psychol. ***11***: 605.
36. Wood, G., Wright, D.J., Harris, D., et al. (2021). Testing the construct validity of a soccer-specific virtual reality simulator using novice, academy, and professional soccer players. Virtual Reality ***25***: 43–51.
37. Witmer, B.G., and Singer, M.J. (1998). Measuring presence in virtual environments: A presence questionnaire. Presence ***7***: 225–240.
38. Slater, M., and &Wilbur, S. (1997). A framework for immersive virtual environments (FIVE): Speculations on the role of presence in virtual environments. Presence. (Camb). ***6***: 603–616.
39. Vignais, N., Kulpa, R., Brault, S., et al. (2015). Which technology to investigate visual perception in sport: Video vs. virtual reality. Hum. Mov. Sci. ***39***: 12–26.
40. Hochmitz, I., and Yuviler-Gavish, N. (2011). Physical fidelity versus cognitive fidelity training in procedural skills acquisition. Hum. Factors ***53***: 489–501.
41. Lathan, C.E., Tracey, M.R., Sebrechts, M.M., et al. (2002). in Handbook of Virtual Environments 443-454 (CRC Press).
42. Alexander, A.L., Brunyé, T., Sidman, J., et al. (2005). From gaming to training: A review of studies on fidelity, immersion, presence, and buy-in and their effects on transfer in pc-based simulations and games. DARWARS Training Impact Group ***5***: 1–14.
43. Wichmann, F.A., and Hill, N.J. (2001). The psychometric function: I. Fitting, sampling, and goodness of fit. Percept. Psychophys. ***63***: 1293–1313.
44. Audiffren, J. (2021). Dichotomous optimistic search to quantify human perception. International Conference on Machine Learning: 414–424.
45. Audiffren, J., and Bresciani, J.P. (2022). Model Based or Model Free? Comparing Adaptive Methods for Estimating Thresholds in Neuroscience. Neural Comput. ***34***: 338–359. ISSN: 1530-888X (Electronic) 0899-7667 (Linking).
46. Pearce, S.P. (2000). The Autobiography (Headline Book Publishing).
47. Owen, M. (2005). Off the Record: My Autobiography (Collins Willow).
48. Eubank, M., and Collins, D. (2000). Coping with pre- and in-event fluctuations in competitive state anxiety: A longitudinal approach. J. Sports Sci. ***18***: 121–131.
49. Jordet, G., and Hartmen, E. (2008). Avoidance Motivation and Choking Under Pressure in Soccer Penalty Shootouts. J. Sport Exerc. Psychol. ***30***: 450–457.
50. Navarro, M., Miyamoto, N., van der Kamp, J., et al. (2012). The effects of high pressure on the point of no return in simulated penalty kicks. J. Sport Exerc. Psychol. ***34***: 83–101.
51. Jordet, G., Hartman, E., Visscher, C., et al. (2007). Kicks from the penalty mark in soccer: The roles of stress,skill, and fatigue for kick outcomes. J. Sports Sci. ***25***: 121–129.
52. Wood, G., and Wilson, M.R. (2012). Quiet-eye training, perceived control and performing under pressure. Psychology of Sport and Exercise ***13***: 721–728.
53. Wood, G., Jordet, G., and Wilson, M.R. (2015). On winning the “lottery”: psychological preparation for football penalty shoot-outs. J. Sports Sci. ***33***: 1758–1765.
54. Jordet, G., Gemser, M.E., and Lemmink, K. (2006). Perceived control and anxiety. International. Journal of Sport Psychology ***37***: 281–298.
55. Guadagnoli, M.A., and Lee, T.D. (2004). Challenge point: a framework for conceptualizing the effects of various practice conditions in motor learning. J. Mot. Behav. ***36***: 212–224.
56. Gray, R. (2017). Transfer of training from virtual to real baseball batting. Front. Psychol. ***8***: 2183.
57. Microsoft. HoloLens 2 Documentation. https://learn.microsoft.com/en-us/hololens/.

ACKNOWLEDGMENTS

This work was supported by the University of Fribourg. The authors would like to thank the managers and trainers of FC Luzern and FC Basel, with special thanks to Christian Schmidt, as well as all players who participated in the experiment.

AUTHOR CONTRIBUTIONS

J.L.B., J.A., T.L.N., and J.P.B. conceived the research idea and designed the study. J.L.B., J.A., and T.L.N. developed the software. A.A., D.S., and G.W. performed the experiments. J.L.B., J.A., and J.P.B. analyzed the data and wrote the manuscript. All authors reviewed and edited the manuscript.

DECLARATION OF INTERESTS

The authors declare no competing interests.

SUPPLEMENTAL INFORMATION

It can be found online at https://doi.org/10.1016/j.xinn.2024.100584.

The Innovation, Volume *5*

Supplemental Information

It's not all in your feet: Improving penalty kick performance with human-avatar interaction and machine learning

Jean-Luc Bloechle, Julien Audiffren, Thibaut Le Naour, Andrea Alli, Dylan Simoni, Gabriel Wüthrich, and Jean-Pierre Bresciani

Supplemental material

The SimulKick application

SimulKick was developed in the C# programming language with the Unity 3D real-time engine and the Microsoft's MRTK augmented reality toolkit. The frequency of the physics engine of SimulKick was set to 100 Hz. During the experiment, SimulKick continuously communicated with the KickManager application via Wi-Fi (using UDP communication together with the JSON data-interchange format). KickManager was developed in the Java programming language, and used by the operator to manage player sessions and upload them to SimulKick. The KickManager application was also used to transmit real-time data from the LIDAR to the SimulKick application, in particular to indicate whether the ball was on the penalty mark. Both the SimulKick and the KickManager applications were developed in our lab.

Animation of the holographic goalkeeper

To create the animations of the holographic goalkeeper, a goalkeeper who plays in the Swiss first league came to our lab for a two-hour motion capture session. During this session, we performed kicks with a real ball to mimic penalty kicks, and the goalkeeper performed dives and saves, executing all movements usually performed by the goalkeeper on the pitch when trying to stop a penalty kick. The goalkeeper was equipped with 49 infrared reflective markers, and his movements were captured using 12 infrared cameras (OptiTrack system, NaturalPoint, Inc.) at a sampling rate of 120 Hz. The skeleton and its movements were 'reconstructed' by the Motive software (NaturalPoint, Inc.). This reconstruction consisted in creating a skeleton consistent with the morphology of the physical

goalkeeper and in updating the skeleton rotations based on the 3D displacements of the markers over time. A 3D mesh of the avatar based on the morphological dimensions of the physical goalkeeper was then created with Fuse (Adobe). Overall, the whole process was very similar to that used to create and animate characters for video games or movies like Avatar.

Parameters of the Experiment

The time at which the dive was triggered could change from trial to trial based on two factors, namely the estimated time before foot-ball contact and the estimated level of performance of the player at this stage of the training. Before detailing how these parameters were estimated, we will define the three parameters which characterized each trial:

The parameter SIDE corresponded to the initial direction of the kick, with two possible values, namely SIDE $= \mathbf{L}$ (left) or SIDE $= \mathbf{R}$ (right). This parameter was the only one communicated to the player before each trial. The second parameter, KICK, corresponded to the necessity (or not) for the player to redirect the kick, noted KICK $= \mathbf{RE}$ (redirection required) or KICK $= \mathbf{NO}$ (no redirection required). This second parameter depended on both the initial target position and the side of the dive of the holographic goalkeeper. The third parameter was DEL, which corresponded to the delay/lag between the onset of the dive of the holographic goalkeeper and foot-ball contact (i.e., kick of the player). Note that the time before foot-ball contact could obviously not be enforced during the experiment. Instead, the run-up of each player was modeled using his previous run-ups, and for each trial, the time to foot-ball contact was estimated using the modeled run-up. For each player, the run-up was modeled with a time/radius mapping, i.e., by associating the time to foot-ball contact to the distance to the penalty mark

(sampling from 0 cm to 200 cm, using 1 cm steps). When the player made a run-up to take a penalty kick, SimulKick used the previously modeled run-ups to estimate in real-time the remaining run-up time and trigger the goalkeeper's dive as close as possible to the predicted time.

After each kick, we recorded the actual time interval (TIME) between the dive of the goalkeeper and the kick, as well as the result (RES) of the kick. The value of this latter parameter could be either **F** (when the ball missed the goal), **W** (when the ball was kicked towards the wrong side of the goal, namely the one where the goalkeeper dove), or **G** (when the ball was kicked towards the correct side of the goal, namely the open one). When RES $=$ **F** or RES $=$ **W** (see also player anticipation below), or when the difference between TIME and DEL was too large (see below), the kick was considered incorrect and was therefore repeated later in the session.

Measured Time and Predicted Delay. The predicted delay DEL and the actually recorded time TIME were never identical. Specifically, our algorithm predicted the time to foot-ball contact, but small, ecological variations of the player's run-up obviously affected this prediction. We modeled this phenomenon by assuming that :

$$\begin{aligned} \text{TIME} &= \text{DEL} + \epsilon \\ \epsilon &\sim \mathcal{N}(0, \varepsilon^2) \end{aligned} \tag{1}$$

where ϵ is a centered Gaussian random variable of variance ε^2, which encodes these small variations. This had two consequences on our model. First, we used the observed time TIME instead of DEL to assess the player's performance, as this is the value that was actually tested in the experiment. Second, we only used trials in which the difference was small enough, namely two standard deviations of the

Gaussian random variable, defined as 2ε. For larger variations, we assumed that the run-up of the player had an unusual pattern (for instance, the player stopped and waited for the goalkeeper to jump), making this trial significantly different in nature from the other trials. Such 'aberrant' trials were set aside during the analysis, and another occurrence of this very trial (i.e., with the same parameters) was repeated at a random point later during the session.

The other type of trial that was considered invalid was the anticipated redirection, which occurred when KICK $= \mathbf{NO}$ but RES $= \mathbf{W}$. In this case, the player was supposed to kick the ball towards the initially indicated target/side, but instead redirected the kick, and this even though it was not required because the goalkeeper actually dove to the opposite side of the goal. This behavior is antithetical to the main objective of the experiment, which was designed to test and train the players' ability to redirect the kick as late as possible in the run-up to the ball. Therefore, these trials were considered invalid and repeated at a random later point in the session. Also, the proportion of trials with anticipated redirection was used to adjust the level of performance of the player with KICK $= \mathbf{RE}$. This is because if the player anticipates the redirection, the task becomes menial and the results irrelevant. This proportion was used as a guess rate[44] in our model (see below).

Player Performance and Model

In order to provide personalized training parameters and to measure the evolution of a player's performance, our learning algorithm builds an internal representation of the performance of each player.

To this end, the kicks were split in four categories, depending on the SIDE and KICK values, i.e., the initial target side and whether the kick had to be redirected

or not.

The assessment of the result as well as the modeling of the player's performance were conducted independently for each side (SIDE $= \mathbf{L}$ and SIDE $= \mathbf{R}$). Therefore, and for the sake of brevity, SIDE is omitted in the following.

Modeling kicks for which no redirection was required. Supplemental Figure 4 depicts the Bayesian Network used to model the performance for non-redirected kicks (i.e., no redirection required). We modeled the probability of outcome of a kick without redirection (KICK $= \mathbf{NO}$) as follows:

$$\mathbb{P}(\text{RES} = \mathbf{F}|\text{KICK} = \mathbf{NO}) = p_N^{\mathbf{F},\mathbf{NO}}$$

$$\mathbb{P}(\text{RES} = \mathbf{W}|\text{KICK} = \mathbf{NO}) = (1 - \underbrace{\mathbb{P}(\text{RES} = \mathbf{F}|\text{KICK} = \mathbf{NO})}_{\text{Failure }\mathbf{NO}}) \times p_N^{\mathbf{RE}}$$

$$\mathbb{P}(\text{RES} = \mathbf{G}|\text{KICK} = \mathbf{NO}) = (1 - \underbrace{\mathbb{P}(\text{RES} = \mathbf{W}|\text{KICK} = \mathbf{NO})}_{\text{Anticipation}}) \times (1 - \underbrace{\mathbb{P}(\text{RES} = \mathbf{F}|\text{KICK} = \mathbf{NO})}_{\text{Failure }\mathbf{NO}})$$

where $p_N^{\mathbf{F},\mathbf{NO}}$ and $p_N^{\mathbf{RE}}$ are two unknown parameters that are assumed to be constant during a session N. The network first evaluates if the player missed the kick, with $\mathcal{B}(p_N^{\mathbf{F},\mathbf{NO}})$, a Bernoulli random variable of mean $p_N^{\mathbf{F},\mathbf{NO}}$. If the player did not miss the kick, the network then evaluates if the player anticipated the (re)direction of the kick, with an independent Bernoulli random variable of mean $p_N^{\mathbf{RE}}$. The kick was considered valid if the player neither missed nor anticipated.

Importantly, our model assumes that the probability of these events is independent from TIME and DEL. Specifically, when KICK $= \mathbf{NO}$, the player is not supposed to redirect the kick after the goalkeeper dive. Therefore, the task is not harder (resp. easier) for shorter DEL. Moreover, kicking the ball from the penalty mark to a target area that encompasses a third of the goal is a relatively easy task for professional football players. We therefore assumed that $\mathbb{P}(\text{RES} = \mathbf{F}|\text{KICK} = \mathbf{NO})$ should be relatively small, and that all kicks directed

to the wrong side of the goal result from an anticipated redirection of the penalty taker.

Modeling kicks requiring redirection. Supplemental Figure 5 depicts the Bayesian Network used to model the performance for redirected kicks (i.e., redirection required). The probability of scoring when a kick redirection is required (KICK $=$ $\mathbf{RE}$) is assumed to depend on the time between the goalkeeper dive and foot-ball contact. This is modeled by:

$$\mathbb{P}(\text{RES} = \mathbf{G} | \text{KICK} = \mathbf{RE}) = (1 - \underbrace{p_N^{\mathbf{F},\mathbf{RE}}}_{\text{Failure } \mathbf{RE}}) \times \big(\underbrace{p_N^{\mathbf{RE}}}_{\text{Anticipation}} + (1 - p_N^{\mathbf{RE}}) \underbrace{\Phi_{\mu_N, \sigma_N}(T_N)}_{\text{Redirection}} \big)$$

$$\mathbb{P}(\text{RES} = \mathbf{F} | \text{KICK} = \mathbf{RE}) = 1 - \mathbb{P}(\text{RES} = \mathbf{G} | \text{KICK} = \mathbf{RE})$$

where $p_N^{\mathbf{F},\mathbf{RE}}$, $p_N^{\mathbf{RE}}$, μ_N, σ_N are four unknown parameters that are assumed to be constant during a session N, while T_N is the TIME value observed for this trial.

In other words, the model works as follows: First, it evaluates whether the player missed the kick, with $\mathcal{B}(p_N^{\mathbf{F},\mathbf{RE}})$, a Bernoulli random variable of mean $p_N^{\mathbf{F},\mathbf{RE}}$. If the kick was not missed, the network then evaluates if the player anticipated redirection, with an independent Bernoulli random variable of mean $p_N^{\mathbf{RE}}$. If the player anticipated redirection, then the kick likely succeeded, because in this case, redirection was planned, which means that no 'late' reprogramming was required. In this scenario, the task was much easier, especially for professional football players. If the player did not anticipate redirection, then the model assesses if the player was able to redirect the kick at the last moment. This probability is equals to $\Phi_{\mu_N, \sigma_N}(T_N)$, i.e., the value of the cumulative distribution function of a Gaussian random variable of mean μ_N and variance σ_N^2, evaluated on time T_N. The choice of this function is discussed below. If the player failed to redirect the kick, then the kick failed (RES $=$ $\mathbf{F}$).

The Redirection Function.. In our model, the choice of the cumulative distribution function

$$\Phi_{\mu_N,\sigma_N}(T_N) = \frac{1}{\sqrt{2\pi\sigma_N^2}} \int_{t=-\infty}^{T_N} \exp\left(-\frac{(t-\mu_N)^2}{2\sigma_N^2}\right) dt \quad (2)$$

for the redirection probability highlights the link between this particular problem and psychometric functions. In particular, the redirection task satisfies similar assumptions: it is a continuous, non-decreasing function of time (i.e., the longer the delay, the easier the redirection). In line with this remark, the method used to estimate the parameters of Φ has some similarity to Bayesian optimization techniques commonly used in psychophysics[44]. Similarly, the anticipation and failure rates $p_N^{\mathbf{F},\mathbf{RE}}$, $p_N^{\mathbf{F},\mathbf{NO}}$ and $p_N^{\mathbf{RE}}$ can be seen as variations of the guess and lapse rates, respectively. However, our setting yields a key difference : the intensity of the stimulus (here, the value of the delay) cannot be specified, due to the difference between TIME and DEL discussed before. Therefore, sampling methods such as maximum information[58] cannot be used directly here.

The Model Parameters.. As mentioned before, both directions of initial intent ($\mathbf{L}$ and $\mathbf{R}$) are treated independently in our model. Therefore, each parameter of the model has two values, which are computed on the two different datasets (Left kicks and Right kicks) using the same method. In our model, we used distinct failure rates for KICK $= \mathbf{NO}$ and $\mathbf{RE}$, ($p_N^{\mathbf{F},\mathbf{RE}}$ and $p_N^{\mathbf{F},\mathbf{NO}}$). This is because the two tasks are distinct, and redirecting a kick is harder than not redirecting it. Therefore, it is reasonable to assume that redirected kicks will miss the target more frequently than non-redirected kicks, and thus ($p_N^{\mathbf{F},\mathbf{RE}} > p_N^{\mathbf{F},\mathbf{NO}}$). The anticipation rate $p_N^{\mathbf{RE}}$ was assumed to be independent of the variable KICK because it encodes the intent of the player to redirect the ball before knowing whether it will be required or not (i.e., before observing KICK). Finally, μ_N, σ_N encode the

model inner representation of the player's performance in redirecting the kick. It is easy to see from (2) that μ_N represents the delay at which the player has a 50 % chance of successfully redirecting the kick, while σ_N quantifies how fast the player's performance improves (or worsens) when the delay varies. All parameters are supposed to stay constant within a session, but they might vary between sessions.

Adaptive Sampling

At the beginning of each session, the cumulative distribution function Ψ_N of the current belief about the values of μ_N is produced (see below Evolution of performance). Using Ψ_N, we set DEL_{min} and DEL_{max} as the values of μ that correspond to a 5% and 95% probability, respectively.

$$\begin{aligned}\text{DEL}_{\text{min}} &= \Psi_N^{-1}(0.05)\\ \text{DEL}_{\text{max}} &= \Psi_N^{-1}(0.95)\end{aligned} \tag{3}$$

Intuitively, DEL_{min} (resp. DEL_{max}) represents the lowest (resp. highest) likely delay for which the player may have a 50% chance of successfully redirecting his kicks. Then, the algorithm proceeds to sample 12 equidistant values of DEL that span the $[\text{DEL}_{\text{min}}, \text{DEL}_{\text{max}}]$ interval.

Updating the model

After each session, the different parameters of the model are estimated as follows: First, using the non-redirected kicks, $p_N^{\mathbf{F},\mathbf{NO}}$ and $p_N^{\mathbf{RE}}$ are estimated using the empirical averages. Then, using the redirected kicks, a posterior for $p_N^{\mathbf{F},\mathbf{RE}}, \mu_{N+1}, \sigma_{N+1}$ is computed for usage in the next session. Finally, an estimator of the current performance of the player is computed based on previous

performance and using a Kalman filter. This estimator is then returned to the experimenter as an indicator. Importantly, the estimator is only used for indicative feedback, but neither for the update of the player's performance model, nor for the data analysis. Supplemental Figure 6 depicts the sampling and updating steps.

Linear mixed-effects models

The two main factors were entered into the model as fixed effects, whereas the intercepts for the participants were entered as random effects. Four models were fitted, namely, 1. A model only including the intercept, 2. A model including the intercept and the Session as predictor, 3. A model including the intercept and both the Session and Redirection as predictors, and 4. A model including the intercept, the two factors as predictors and an interaction term. The four models were compared and p-values were obtained using likelihood ratio tests. The degrees of freedom were approximated using the Kenward-Roger method[59]. For each analysis, we also fitted three models including random slopes. The pattern of results was exactly the same whether the models included random slopes or not. However, the Akaike and Bayesian Information Criteria (i.e., AIC and BIC) were higher for the models including random slopes. We therefore do not report the results obtained with these 'overfitted' models.

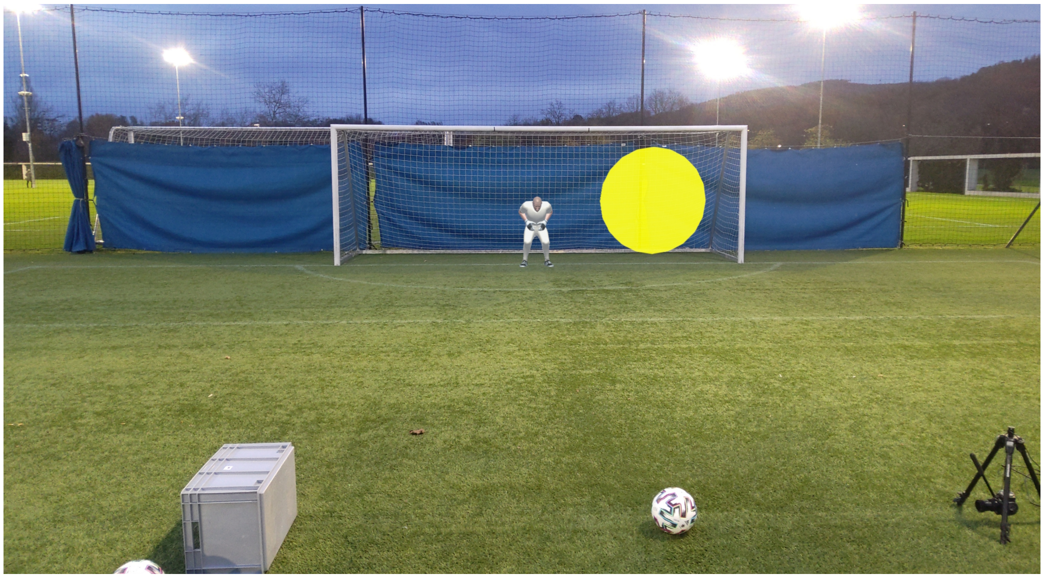

Supplemental Figure 1: Screenshot of a (right-footed) player's view just before starting the run-up to the ball. The holographic goalkeeper and the target area are displayed in the Microsoft HoloLens 2 Augmented-Reality headset. For this trial, the player is instructed to kick the ball to the left side of the goal, i.e., towards the yellow target area. However, if the goalkeeper dives to that side during the run-up, the player should try to redirect the kick to the right (i.e., open) side of the goal.

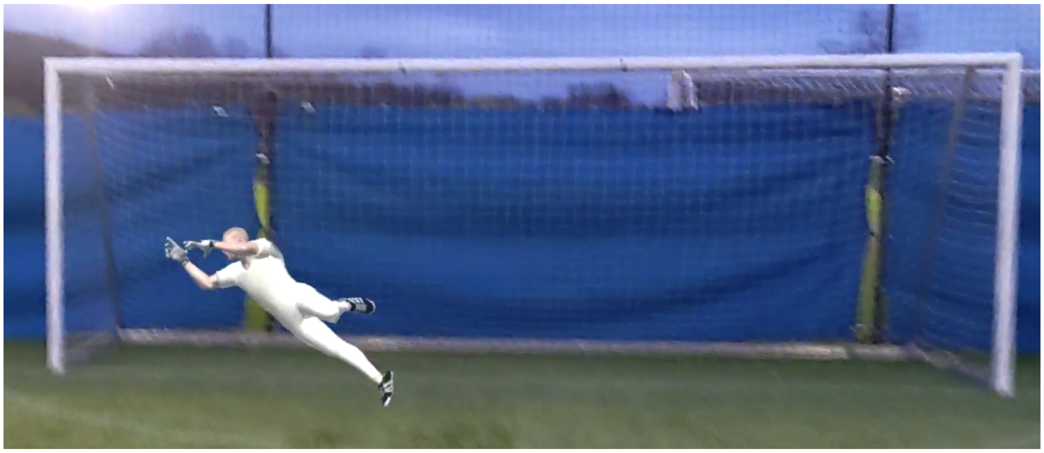

Supplemental Figure 2: The holographic goalkeeper is diving during the run-up of the player to the ball.

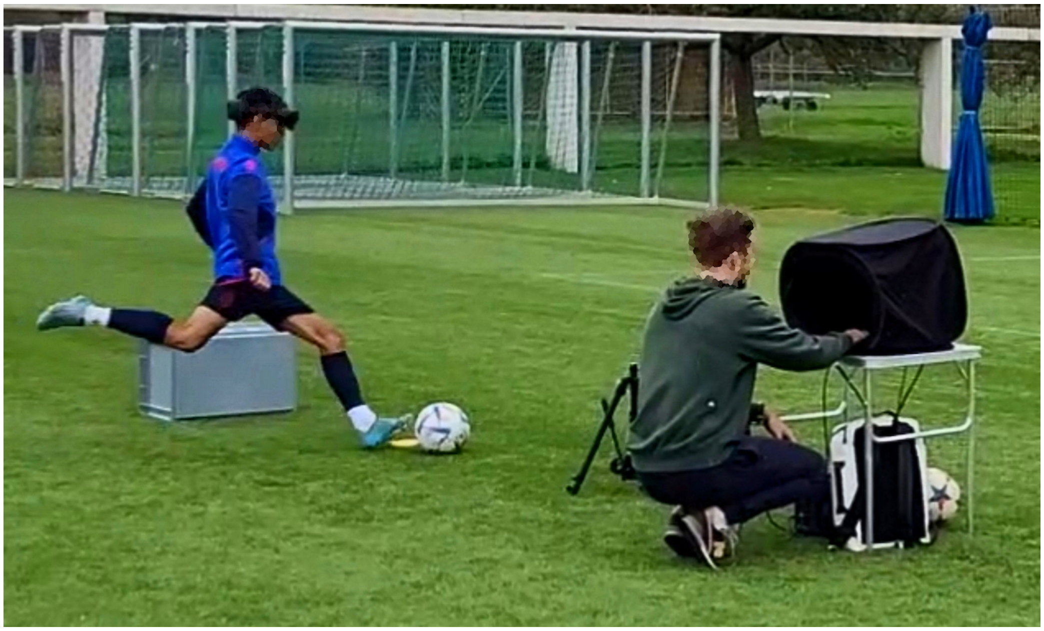

Supplemental Figure 3: A (right-footed) player about to kick the ball at the end of the run-up. The player sees the scene through the Microsoft Hololens 2 headset.

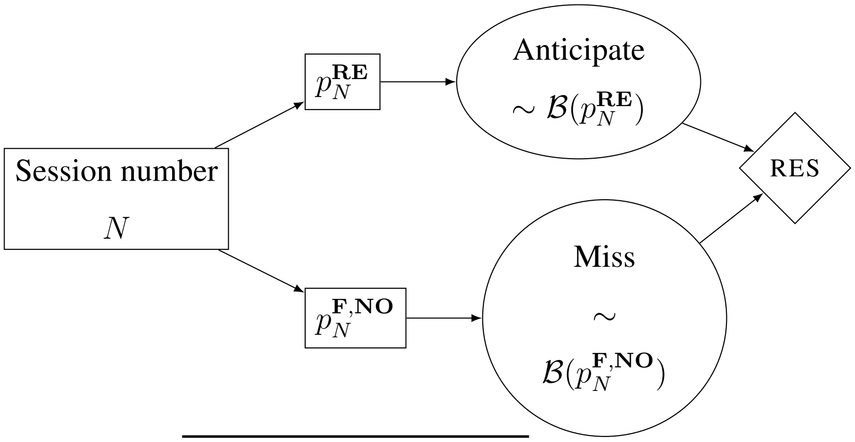

Miss	Anticipate	RES
True	-	**F**
False	True	**W**
False	False	**G**

Supplemental Figure 4: Bayesian Network used for modeling kicks for which no redirection was required. Rectangles represent parameters, ellipses random variables and diamonds the observed variables. Each random variable follows a Bernoulli distribution $\mathcal{B}$, and the relation between the hidden variables and the observed result is summarized in the joint Table.

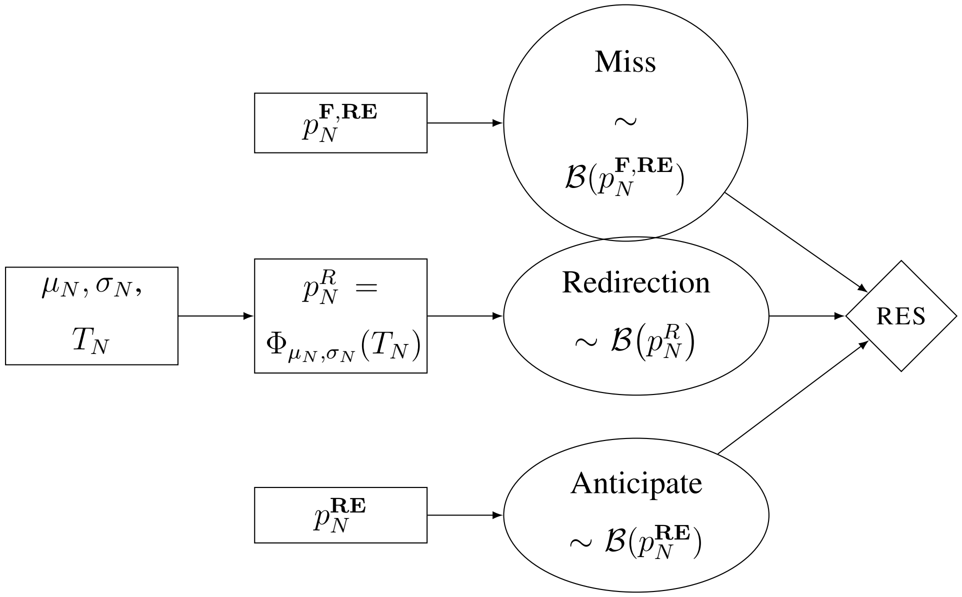

Miss	Anticipate	Redirection	RES
True	-	-	**F**
False	True	-	**G**
False	False	True	**G**
False	False	False	**F**

Supplemental Figure 5: Bayesian Network used for modeling the kicks requiring redirection. The session number N is also a parameter, but it has been removed to improve readability. Rectangles represent parameters, ellipses random variables, and diamonds the observed variables. Each random variable follows a Bernoulli distribution $\mathcal{B}$, and the relation between the hidden variables and the observed result is summarized in the joint Table.

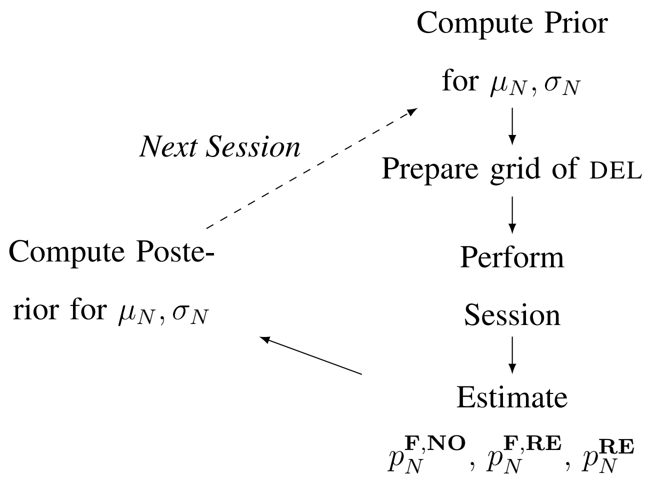

Supplemental Figure 6: Summary of the sampling and update of the model.
